# Enhancing Empathic Accuracy: Penalized Functional Alignment Method to Correct Temporal Misalignment in Real-Time Emotional Perception

**DOI:** 10.1017/psy.2025.10040

**Published:** 2025-09-05

**Authors:** Linh H. Nghiem, Jing Cao, Chrystyna D. Kouros, Chul Moon

**Affiliations:** 1 School of Mathematics and Statistics, University of Sydney, Sydney, NSW, Australia; 2 Department of Statistics and Data Science, Southern Methodist University, Dallas, TX, USA; 3 Department of Psychology, Southern Methodist University, Dallas, TX, USA

**Keywords:** cognitive study, functional data analysis, regularization, square root velocity function, warping function

## Abstract

Empathic accuracy (EA) is the ability to accurately understand another person’s thoughts and feelings, which is crucial for social and psychological interactions. Traditionally, EA is assessed by comparing a perceiver’s moment-to-moment ratings of a target’s emotional state with the target’s own self-reported ratings at corresponding time points. However, misalignments between these two sequences are common due to the complexity of emotional interpretation and individual differences in behavioral responses. Conventional methods often ignore or oversimplify these misalignments, for instance by assuming a fixed time lag, which can introduce bias into EA estimates. To address this, we propose a novel alignment approach that captures a wide range of misalignment patterns. Our method leverages the square-root velocity framework to decompose emotional rating trajectories into amplitude and phase components. To ensure realistic alignment, we introduce a regularization constraint that limits temporal shifts to ranges consistent with human perceptual capabilities. This alignment is efficiently implemented using a constrained dynamic programming algorithm. We validate our method through simulations and real-world applications involving video and music datasets, demonstrating its superior performance over traditional techniques.

## 1 Introduction

The ability to perceive and understand the emotions and thoughts of others, broadly referred to as empathy, plays an important role in human society by facilitating cooperation and social cohesion (De Waal, 2008). While empathy encompasses multiple components, including sharing in another’s emotional experience and concern for others, empathic accuracy (EA) refers to the specific skill of accurately inferring what another person is thinking and feeling in a given moment (Ickes, 1997). EA is typically measured behaviorally by comparing a perceiver’s rating of a target’s emotional state to the target’s own self-reported emotional experience. Given its importance to social interactions and quality of life, EA has become a focal point of research across various fields. For example, in social science, EA’s role was examined in developing and maintaining healthy social relationships (Sened et al., 2017). In clinical research, EA has been used as an index to differentiate individuals with certain psychiatric disorders from healthy controls (Lee et al., 2011). However, the validity of these studies critically depends on the quality and accuracy of EA measurement.

**Figure 1:**
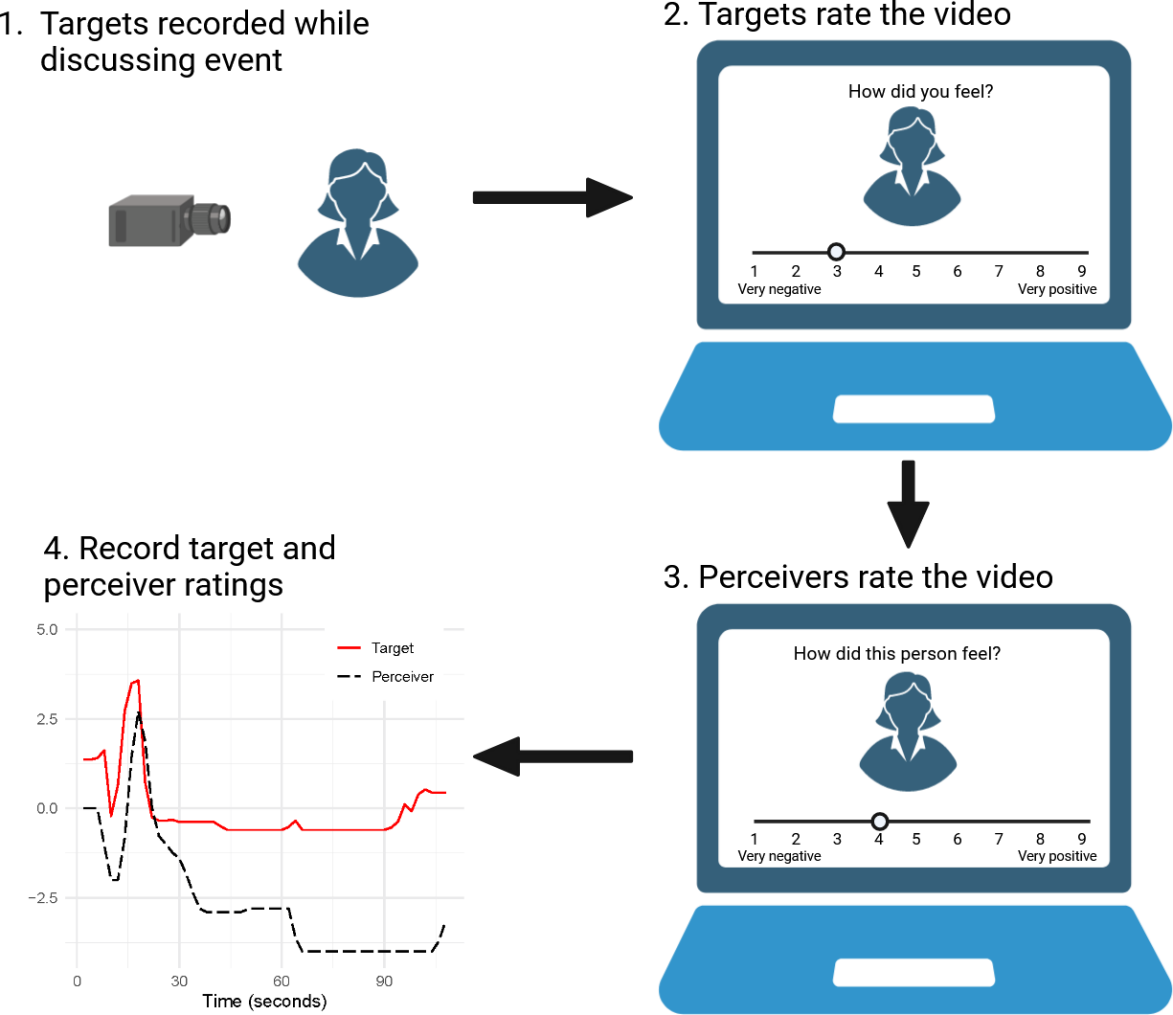
Example of real-time EA data collection procedure.

There are two types of studies commonly used to examine EA. One is the non-real-time EA study design, where perceivers provide their response to stimuli after the stimuli have been conducted. The outcome of their overall empathy can be the categories of emotion (e.g., happiness, anger, sadness, etc.) or the extent of emotion on a Likert-type scale (Ekman, 1992; Schweinle et al., 2002). The other EA study design is the real-time assessment of perceivers’ empathy on an audio or video stimuli without pausing (i.e., the recorded affective states of targets) (Jospe et al., 2020; Zaki et al., 2008), where perceivers provide continuous feedback on their perceptions of the target’s emotional state while the stimuli is unfolding. Illustrated in Figure 1, social targets varying in trait emotional intensity were videotaped while discussing emotional autobiographical events. Perceivers watch these videos and report the perceived emotions every two seconds using, for example, a 9-point Likert scale (e.g., 1 = extremely negative; 9 = extremely positive). Compared with the non-real-time EA studies, the real-time design provides more granular information on the dynamic nature of empathy in everyday interactions and detects subtle changes in emotional responses that might be missed in non-real-time assessments.

In this article, we focus on analyzing data from real-time EA study designs. For such designs, correlational analysis (Mackes et al., 2018; Zaki et al., 2009, among others) is a predominant statistical method for examining EA. This approach computes a monotonic transformation of the Pearson correlation between the observed perceivers’ responses with targets’ self-reported emotion rating. Linear models have also been introduced to investigate the influence of additional factors or unobserved variables on EA. For example, Tabak et al. (2022) proposed a latent variable model that decomposes EA into three separate dimensions: bias, discrimination, and variability. Bias measures the systematic difference between perceiver’s ratings and target’s ratings; discrimination measures perceiver’ sensitivity in relation to target’s ratings; and variability measures the variance of random error in perceiver’s perceptions.

A key assumption in traditional correlational and linear model analyses of EA is that perceivers’ and targets’ rating sequences are perfectly aligned—that is, a perceiver’s rating at a given time point is directly compared to the target’s rating at that same moment. However, this assumption often fails in practice due to the complex cognitive processes involved in interpreting another person’s emotional state and the time required to produce a behavioral response. Scherer’s multi-stage model of emotion decoding Scherer (2003) highlights how perceivers actively interpret dynamic cues such as facial expressions, gestures, and vocalizations to infer emotions, a process that naturally introduces temporal delays. Additionally, the act of recording a response, such as pressing a key or moving a joystick, can vary in duration, further contributing to misalignment. To address these issues, we posit that each perceiver has an underlying latent rating that reflects their true empathic understanding, independent of these timing distortions.

**Figure 2:**
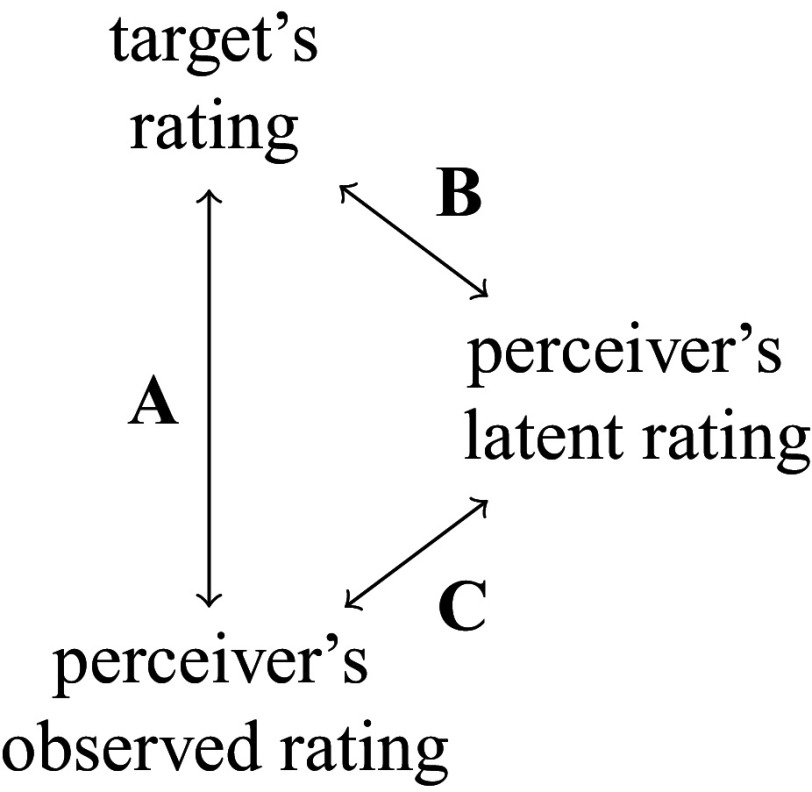
Illustration of different relations between a target’s rating, a perceiver’s latent rating, and the perceiver’s observed rating. Discrepancy A denotes the difference between a target’s rating and a perceiver’s observed rating. Disagreement B captures the inconsistency between target’s rating and perceiver’s latent rating, which is the true focus of EA. Misalignment C refers to the divergence between perceiver’s observed rating and their latent rating, often due to distortions in expressing their internal judgment. Discrepancy A can arise from both Disagreement B and Misalignment C. Most conventional EA methods mistakenly assess Discrepancy A, thereby conflating measurement error with genuine empathic inaccuracy.

Figure 2 illustrates the relations between a target’s rating, a perceiver’s latent rating, and the perceiver’s observed rating. Discrepancy A, the difference between the target’s rating and the perceiver’s observed rating, can arise from two sources: Disagreement B, which reflects the true empathic inaccuracy between the target’s rating and the perceiver’s latent rating, and Misalignment C, which captures the temporal mismatch between the perceiver’s latent and observed ratings. Crucially, EA is intended to measure a perceiver’s ability to correctly infer another person’s emotional state—that is, to quantify Disagreement B—not the speed or timing of their response. A perceiver who accurately identifies a target’s emotion, even with a slight misalignment, should not be penalized as less empathically accurate. However, traditional EA methods often ignore Misalignment C, relying solely on comparisons between the target’s and perceiver’s observed ratings (i.e., measuring Discrepancy A). This could potentially lead to biased estimates of EA and inflated variability due to even minor timing discrepancies. Our work addresses this limitation by explicitly correcting for Misalignment C, thereby yielding more accurate estimates of Disagreement B and preserving the psychological validity of EA assessments.

**Figure 3:**
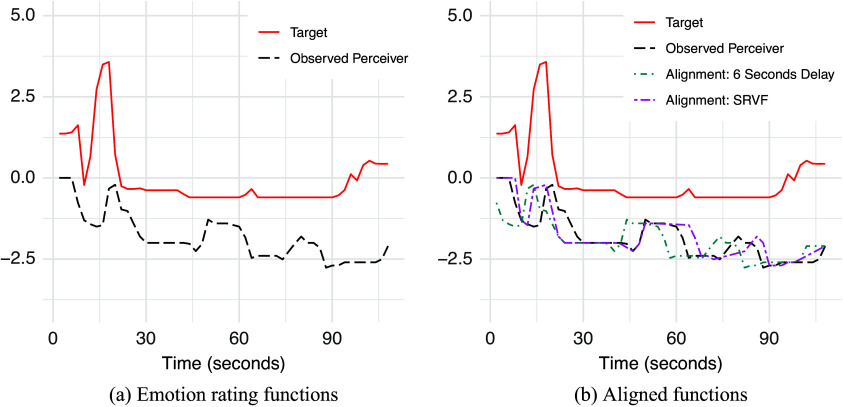
(a) An example of misaligned rating sequences between a perceiver and a target. The solid red line represents the target’s ratings and the black dashed perceiver’s ratings. (b) Aligned ratings for the perceiver. The green dashed line shows the 6-second delay adjustment, and the purple dashed line shows the aligned ratings using the penalized SRVF representation.

Note that common approaches to address misalignment in EA studies involve introducing a fixed response delay, assuming consistent emotional expression patterns across individuals. This method shifts perceivers’ response time series backward by a predetermined amount (Huang et al., 2015; Khorram et al., 2019; Nicolle et al., 2012). However, Scherer (2003) countered this assumption, arguing that emotional expressions are diverse and context-dependent. In addition, a review of event-related potential (ERP) studies spanning 40 years found that emotional stimuli elicit differences in neural processing speed based on valence and arousal level Olofsson et al. (2008). The findings suggest that stimuli with higher motivational relevance receive priority in neural processing. Consequently, the misalignment between perceivers’ and targets’ ratings is more complex than a simple, fixed time shift applied to all participants. It would be inappropriate to apply a fixed time delay in EA studies, as it fails to account for the variability in emotional processing across different moments. Figure 3a illustrates an example of misalignment between perceiver and target ratings in an EA study (Devlin et al., 2014). While a delay in perceiver responses is evident, it is not the sole cause of misalignment. For instance, the perceiver’s prolonged sustained response from 10 to 15 seconds, in contrast to the target’s brief dip at 10 seconds, highlights the complex nature of these discrepancies.

To accommodate a wider range of misalignment patterns beyond simple time shifts, time series alignment methods aim to preserve key structural features in the data, such as peaks and valleys, ensuring more accurate analysis. One widely used technique is dynamic time warping (DTW), which aligns time series by stretching or compressing the time axis to match similar patterns (Berndt & Clifford, 1994; Sakoe & Chiba, 1978). However, DTW can sometimes introduce distortions by forcing unnatural alignments between sequences (Marron et al., 2015; Srivastava et al., 2011; Zhao et al., 2020). To mitigate this, smoothness penalties have been proposed (Ramsay & Silverman, 2005). However, it may also lead to biased alignments (Guo et al., 2022). Alternatively, landmark-based methods align time series by identifying and matching distinctive features like peaks and valleys (Kneip et al., 2000). While potentially effective, these methods are highly sensitive to noise and may lose important information due to the discretization of continuous functions into a limited set of landmarks (Marron et al., 2015; Wang & Gasser, 1997). Moreover, such approaches are ill-suited for real-time emotion rating data in EA studies, where there is no clear consensus on the number or the location of meaningful landmarks.

Due to the high-frequency nature of the observed EA rating data, we treat each observed curve as a sample path of a continuous function in the time domain, i.e., functional data. Such an approach of representing high-frequency data as functional is common in the literature (Kokoszka & Reimherr, 2017). From this perspective, misalignment between two observed ratings could be explained by a smooth warping function that distorts the time domain of the perceiver relative to that of the target. Hence, the target and the perceiver’s rating functions can be aligned by estimating this smooth warping function from the observed data, for example, by minimizing an 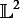 distance between the target and the estimated aligned response function (Ramsay & Li, 1998). Recently, the square root velocity function (SRVF) representation has been employed for aligning functions (Srivastava et al., 2011), and has been increasingly applied across various fields, including biology, medicine, geology, and signal processing (Bharath et al., 2018; Laga et al., 2014; Mitchell et al., 2025; Su et al., 2014; Zhao et al., 2020). As we will review in Section 2, this SRVF representation leverages the Fisher–Rao metric’s invariance property, and enables a consistent separation of horizontal component (also known as phase) from vertical component (also known as amplitude) of functions, making visualization and summarizing variability in functional datasets more effective (Xie et al., 2017).

Building upon the SRVF-alignment framework, this article introduces a novel penalized SRVF-based alignment method for unsynchronized rating sequences in EA studies. Our approach introduces both practical and methodological innovations. Practically, it is the first method in EA research to accommodate a wide range of misalignment patterns (e.g., delays, compressions, and stretches), moving beyond the limitations of fixed-delay adjustments. Methodologically, we incorporate a novel penalty term that constrains temporal shifts within bounds consistent with human perceptual capabilities, thereby preventing excessive or unrealistic alignments (Gunes & Pantic, 2010; Levenson, 1988; Mariooryad & Busso, 2014; Ringeval et al., 2015). This is important because not all temporal discrepancies should be corrected; some may reflect genuine empathic inaccuracy rather than misalignment. To address this, our penalized alignment method selectively adjusts only short-term misalignments—those occurring within a few seconds—treating them as Misalignment C (as shown in Figure 2). In contrast, larger discrepancies, which may indicate a lack of empathic understanding (Disagreement B), are preserved.

To highlight the contribution of our method, Figure 3b compares the proposed penalized SRVF method with a fixed 6-second delay adjustment. Although the 6-second delay adjustment aligns the peaks between the two sequences, it, unfortunately, eliminates the brief 5-second sustain at the start of the perceiver’s sequence, which originally matched up with the target’s self-rating sequence. In contrast, the proposed penalized SRVF-based method has aligned the peaks while keeping the initial sustain in the perceiver’s sequence in place, demonstrating its flexibility in handling complex misalignment patterns. By enabling a more precise alignment, our method yields a more accurate estimation of EA, avoiding the pitfalls of underestimation when misalignment is ignored and overestimation when no penalty is applied.

The remainder of the article is structured as follows. Section 2 provides background information on EA and existing alignment methods. The proposed methodology is detailed in Section 3. To evaluate the proposed method, Section 4 presents a simulation study and comparisons to alternative approaches. Real-world applications of assessing EA in social and music contexts are explored in Section 5. Finally, Section 6 offers a discussion of the findings and concludes the article.

## 2 Background

### 2.1 Elastic functional data analysis

Functional data often exhibit both vertical and horizontal differences, where the latter is known as phase variation and characterized by misaligned geometric features such as peaks and valleys in the time domain (Tucker et al., 2014; Wallace et al., 2014; Wu & Srivastava, 2014). Let 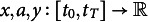 be the function of the target rating, the function of the perceiver’s latent rating, and the function of the perceiver’s observed rating, respectively. To account for both vertical and horizontal difference between these two functions, we assume a data generation process that 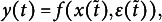 where 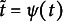 , 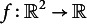 is a link function, 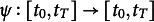 is a time warping function, 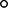 denotes the composition operator, and 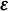 is a random noise function. Essentially, the process of generating the perceiver’s observed rating *y* is decomposed into a transformation step and a warping step, as depicted in the following expression (2.1). 
(2.1)

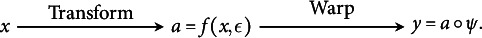



Note that in this transformation step, the link *f* matches the target function *x* at a time point *t* to the perceiver’s latent function *a* at the same time *t*. This correspondence is distorted by a warping function 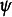 in the second warping step, so the target function *x* at time *t* now is matched to the perceiver’s observed function *y* at 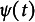 . The warping function 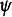 is usually assumed to belong to the set of smoothing functions 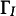 , where 

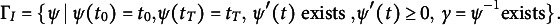



Relating this process to Figure 2, the transformation step models the Disagreement B, while the warping step models the Misalignment C, and the Discrepancy A accumulates both steps together. In the context of measuring EA, the perceiver’s latent function *a* represents a rating from the perceiver that is aligned with *x*, i.e., that *can be compared with the target x point-to-point in time*, and a measure of EA is a similarity measure between *x* and *a*.

The data generation process (2.1) motivates the following workflow for quantifying EA. Because 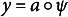 , we can write 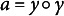 , where the inverse warping function 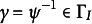 is assumed to exist since 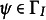 . Hence, we first conduct an alignment step to obtain an estimated inverse warping function 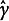 and an estimated latent function 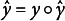 from the observed target and perceiver functions *x* and *y*. Then, we could estimate EA by a similarity measure between *x* and 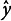 .

Since misalignment between two functions is inherently related to the difference in how fast they move, a common way to conduct the alignment step is to compare how these functions change over time, which is mathematically described by their corresponding first derivative. Therefore, the general idea of the SRVF-based alignment methods is to minimize the distance between the first derivative of the target *x* and the estimated function 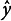 . We briefly review the formulation of the SRVF representation here, where more details can be found in Srivastava & Klassen (2016).

For any absolute continuous function 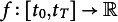 , the SRVF of *f* is the function 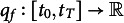 , 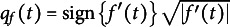 , where 
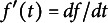
 . As described in the previous paragraph 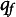 , this SRVF is defined based on the first derivative 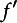 ; the specific form of 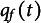 is motivated to keep its norm unaffected by the warping, which is useful to separate a function into its amplitude and phase component (Srivastava & Klassen, 2016). Specifically, if *f* is warped by 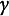 , the corresponding SRVF of 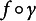 becomes 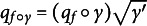 , but the squared 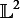 norm is preserved 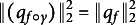 . Let 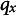 and 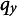 be the SRVFs of the target and perceiver functions, respectively. Then, the SRVF-based alignment method aims to find an optimal inverse warping function that minimizes this discrepancy, i.e., 
(2.2)

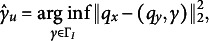

where we write 
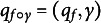
 to ease the notation. The optimal 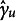 is expected to align two functions so that the transformed function 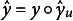 is aligned with *x*. The subscript 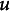 stands for “unpenalized,” meaning the optimal 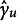 is not subject to any other constraint than being in the space 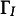 . This unpenalized alignment has been implemented in the fdasrvf packages (Tucker, 2025) in both R and Python. After conducting the alignment step, in addition to the EA measure obtained by computing a similarity metric between 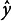 and *x*, we can also quantify the amount of warping made by each perceiver in relative to the target by a Fisher–Rao phase distance between the estimated warping 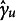 and the identity warping 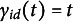 as 
(2.3)

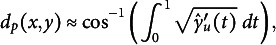

which is a proper metric distance on the set 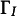 (Srivastava & Klassen, 2016).

### 2.2 Unpenalized SRVF leads to over-alignment

While the SRVF representation leads to several theoretical benefits, one main disadvantage of the unpenalized SRVF for studying EA is that the estimated perceiver function 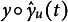 may be overaligned with the target *x* and thus could differ from the perceiver’s latent function *a*. In other words, the unpenalized SRVF not only corrects for the Misalignment C in Figure 2, but also potentially removes inherent temporal Disagreement B.

**Figure 4:**
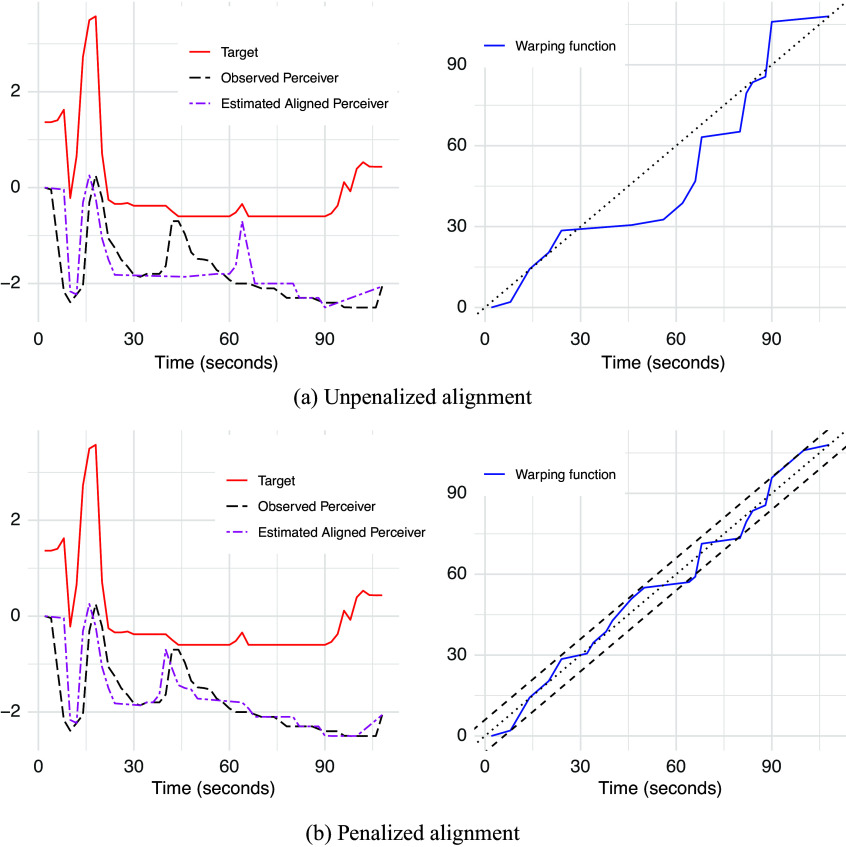
Example target and perceiver’s emotion ratings of Devlin et al. (2014). (Left): target *x* (solid), perceiver’s observed response *y* (dash), and estimated perceiver’s response 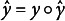 (dot dash). (Right): estimated warping function 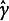 .

Figure 4a shows one example from the study in Devlin et al. (2014) demonstrating the result of the previous SRVF alignment obtained from (2.2). In this study, the continuous ratings were recorded for 108 seconds and averaged over 2-second epochs. The alignment is obtained by using their SRVF representations 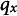 , 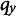 , and 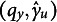 . The estimated inverse warping function 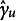 is plotted in the right panel of Figure 4a. When 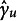 appears above the 45-degree line, it implies that the perceiver’s response is delayed compared to the target, whereas 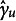 below the 45 degree line indicates that the perceiver’s response precedes the target. While it seems reasonable to expect that the perceiver’s perception of a particular emotion would lag behind the target’s actual expression of that emotion, there is evidence to suggest that people can make anticipatory perceptual judgements, especially when the stimuli are continuous and dynamic. For example, Thornton and Tamir Thornton & Tamir (2017) found that perceivers attend to emotion regularities and can predict up to two emotional transitions into the future. Koster–Hale and Saxe Koster-Hale & Saxe (2013) argued that the brain actively generates expectations about others’ emotions, thoughts, and behaviors (so not just passively reacting to them). They refer to this as “predictive coding.” In Figure 4a, the peak of the perceiver’s response around 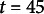 seconds is considered as a response preceding the target’s self-rating around 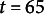 seconds, and it is aligned accordingly by the unpenalized SVRF method.

Therefore, from the left plot of Figure 4a, the unpenalized SRVF method misaligns the peak of the perceiver’s response, occurring at approximately 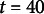 seconds, with the target’s small peak at around 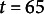 seconds, which is likely just noise. This alignment suggests an improbable scenario, where the perceiver predicts the target’s emotional change 25 seconds in advance. Psychological research has consistently shown that reaction time-delay is limited to a few seconds: 0.5 to 4 seconds (Levenson, 1988), 3 to 6 seconds (Nicolle et al., 2012), 2 to 11 seconds (Mariooryad & Busso, 2014), and 0.48 to 6.24 seconds (Ringeval et al., 2015). By disregarding this inherent limitation, unpenalized SRVF alignment overestimates synchronization between rating sequences, potentially leading to an unrealistic shape of the estimated warping function that exceeds the human exception bounds, and hence biased estimations of perceiver EA levels.

## 3 Method

### 3.1 Penalized elastic functional alignment

Penalized alignment has been proposed to control the amount of alignment (Guo et al., 2022; Mitchell et al., 2025; Wu & Srivastava, 2011) or to achieve smooth alignment (Srivastava & Klassen, 2016). To address the over-alignment issue inherent in the unpenalized SRVF method, an existing solution is to employ a penalized alignment approach by incorporating a penalty term into the unpenalized alignment optimization function (2.2). This results in the following objective function: 
(3.1)

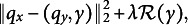

where 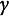 is the inverse warping function, 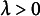 is a penalty parameter, and 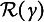 is a penalty function. Several penalty functions have been suggested in the literature, such as 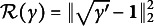 and 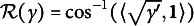 , which are used to measure the differences between the SRVFs of 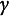 and the identity warping 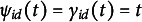 by the squared 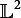 norm and the arc length, respectively, where 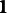 is the constant function with value 1 and 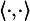 denotes an inner product operator (Srivastava & Klassen, 2016).

The aforementioned penalty functions are inappropriate for current EA research. Primarily, it is challenging to select an optimal data-driven tuning parameter 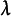 . Common cross-validation procedures that split the data into independent training and test sets do not preserve the geometric features of the data. Second, as reviewed in Section 2.2, psychological research indicates that misalignment in perceiver ratings occurs within a specific temporal window of a few seconds. Existing penalty functions, however, focus on controlling the overall amount of warping, which does not directly translate to constraining alignment at each individual time point as required for EA studies.

To address these limitations, we introduce a novel penalized alignment method that directly incorporates the established temporal boundary for maximum perceiver misalignment as a penalty term. Specifically, we construct the optimal inverse penalized warping function 
(3.2)

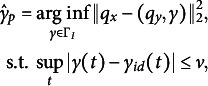

where 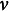 is the predefined upper limit of warping functions, corresponding to the maximum delay or advance observed in the perceivers’ responses. Although the supremum norm limit 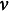 plays the role of a tuning parameter, in practice, we often have prior knowledge about its value based on the research context, unlike the tuning parameter 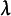 in the existing approach (3.1). Nevertheless, it is useful to perform a sensitivity analysis of the proposed method over a reasonable range of 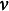 . We denote 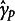 as the estimated inverse warping function of penalized alignment, where the subscript 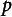 stands for “penalized.” As 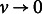 , 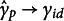 , so that no warping is allowed. On the other hand, if 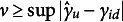 , the constraint in (3.2) is inactive, then 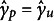 . Consequently, any 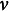 smaller than 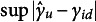 induces a shrinkage effect, pulling the unpenalized warping towards the identity warping function, akin to penalized regression. This interpretable penalty mechanism enables our proposed penalized alignment to mitigate the risk of over-alignment, resulting in more plausible warping estimates and aligned responses.

We note that under the constraint (3.2), the Fisher–Rao phase distance 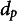 defined by (2.3) is still valid to measure the difference between the phase of two functions, with the exception that 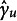 is replaced by 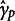 . The proof of Lemma 3.1 is given in Section S1 of the Supplementary Material.

Lemma 3.1. The Fisher–Rao phase distance between *x* and *y* is estimated by 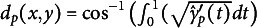 .

### 3.2 Computing the penalized SRVF alignment

A discrete approximation for the solution of the optimization problem specified in (3.2) can be found by using the following dynamic programming algorithm (DPA) (Srivastava & Klassen, 2016). Assume both the SRVF functions of the target and the perceiver 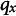 and 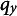 are observed at 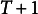 time points, 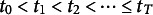 . Without loss of generality, we assume that 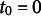 and 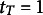 , and that these time points are equally spaced, i.e., 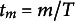 for 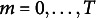 . The inverse warping function 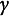 matches the point 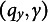 with the point 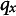 , so 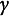 can be viewed as a graph with a collection of points 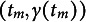 , from 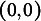 to 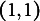 in 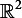 . We assume that within each interval 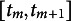 , the function 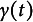 can be approximated by a straight line, so the final estimate for 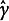 is a piecewise linear path. Since 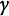 is non-decreasing, the slope of this graph is always strictly between 0 and 90 degrees. Furthermore, the cost function in (3.2) can be approximated by 
(3.3)

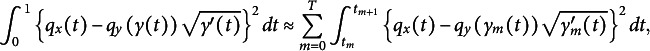

where 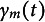 is a straight line connecting 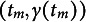 and 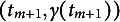 . The function on the right-hand side of (3.3) is additive over the graph, and hence enables the use of DPA. Our goal then is to find an optimal linear piecewise path from 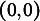 to 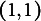 in 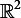 that minimizes (3.3), subject to the constraint that 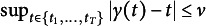 . Using DPA, we can construct this path recursively as follows.

Given a feasible point 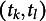 , i.e., 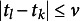 in the graph, let 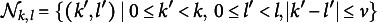 denote the set of all nodes in the graph that are allowed to go to 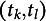 by a straight line. Starting from 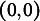 , if we have already determined and stored the cost of reaching nodes in 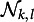 , then the cost of reaching 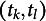 is given by 
(3.4)

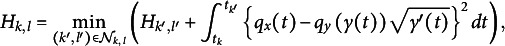

where we initialize 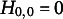 and 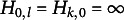 for any 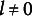 and 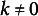 . Let 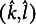 be the nodes that minimize the right-hand side of (3.4) and repeat the process for every possible point 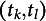 in the graph. Then, the optimal curve 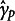 is obtained by connecting all such points using piecewise linear curves. Note that compared to the standard DPA algorithm to align the two functions (Srivastava & Klassen, 2016), we have modified the set of permitted nodes to account for the constraint imposed on the warping function.

The algorithm is summarized in Algorithm 1.

**Figure:**
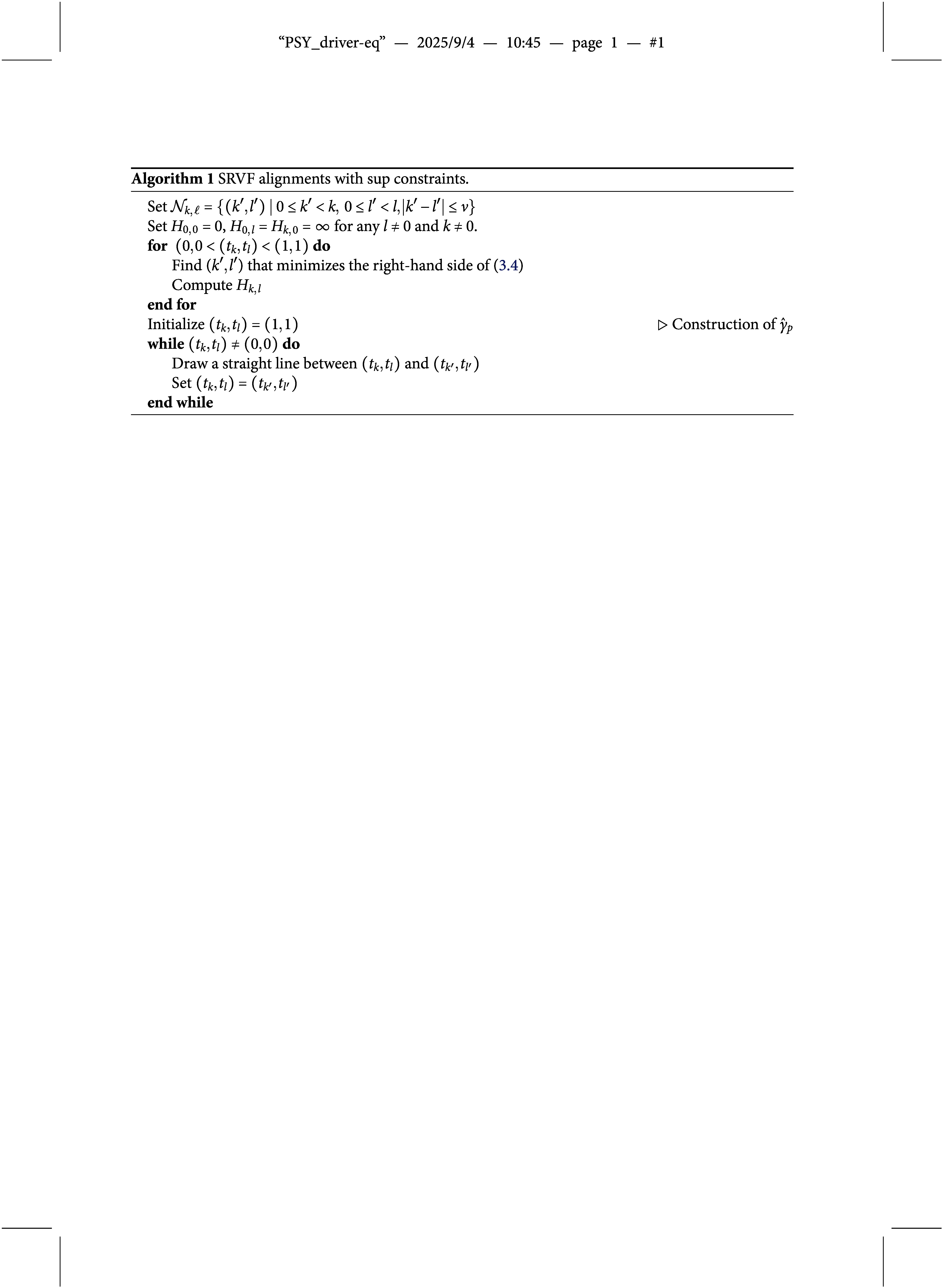

Because the temporal window can vary according to the emotions and the modality (Gunes & Pantic, 2010; Gunes & Schuller, 2013), we used three thresholds of 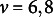 , and 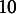 seconds for EA applications in Section 5. Figure 4b shows penalized alignment of the same example presented in Figure 4a, using an upper limit of warping function differences of six seconds ( 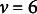 ). Since 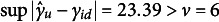 for unpenalized alignment, penalized alignment shrinks the estimated inverse warping function 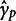 toward the identity warping function. Consequently, the resulting estimated perceiver latent function 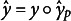 does not exhibit peaks or valleys that deviate from the observed perceiver sequence by more than six seconds.

## 4 Simulation study

To demonstrate the performance of our functional alignment approach, we conducted a number of simulation studies. It is challenging to use real EA data to evaluate functional alignment methods because perceivers’ latent ratings are unknown. However, we generated perceivers’ latent responses from the real target ratings and used them to compare different alignment methods.

### 4.1 Simulation 1

In this simulation, we evaluated the effectiveness of various alignment methods. To approximate the settings in real–data applications, we used the four targets 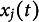 directly derived from the real target data of Devlin et al. (2014) corresponding to four videos: high intensity positive, low intensity positive, high intensity negative, and low intensity negative, for 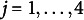 . We smoothed these raw data using the cubic smoothing spline and recorded the functional values for 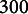 evenly-spaced time points ( 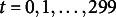 ).

Next, we generated 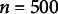 perceivers’ latent responses for each target function 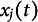 using the following model 

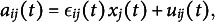

for 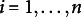 . Here, we set 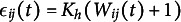 , where 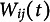 is a one-dimensional Wiener process (i.e., Brownian motion) at time *t* (Mörters & Peres, 2010), 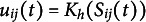 with 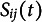 being a standardized random walk at time *t* that is obtained by cumulatively summing the standard normal 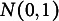 noise and applying a standardization transformation. We denote 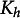 as the Gaussian kernel smoothing with bandwidth *h*, and in this simulation, we used 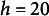 to ensure both 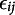 and 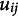 are smooth. Then, we generated the perceiver’s observed response 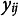 using the perceiver’s latent rating 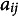 by 
(4.1)

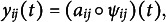

where 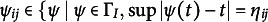 for 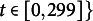 is the warping function and 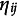 is the true individual upper limit of the warping amount. We first generated the warping functions randomly using the *rgam* function in the R package fdasrvf Tucker (2025), and then rescaled them such that 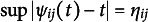 . With that simulation configuration, the true correlation between 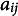 and the target 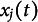 has the mean around {0.65, 0.66, 0.67, 0.66} with standard deviation {0.24, 0.27, 0.23, 0.25} for all 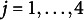 , respectively.

We considered five different methods to align the observed perceiver response to the target, including (1) no alignment, (2) optimal fixed delay, (3) unpenalized SRVF alignment, (4) the squared 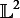 norm penalty SRVF alignment, and (5) our proposed penalized SRVF alignment. Let 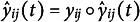 denote the estimated perceiver’s response, where 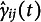 denotes an estimated inverse warping function from one of the above methods. The no alignment option assumes the identity inverse warping 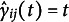 . For the optimal fixed delay method (2), we found the optimum amount of delay 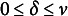 that achieves the smallest 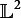 distance between 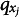 and 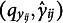 , where 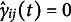 if 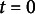 , 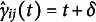 if 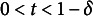 , and 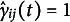 otherwise. The inverse warping functions of the unpenalized and penalized SRVF alignments were obtained by solving (2.2) and (3.2), respectively. The squared 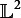 norm penalty SRVF alignment implements the penalty 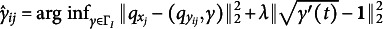 , where 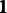 is the constant function with value one (Srivastava & Klassen, 2016). To the best of our knowledge, an optimal method for selecting 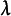 has not been established in the literature, so we implemented the method with 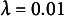 . We leave the investigation of optimal selection strategies for 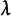 to future research.

In the simulation, we set the alignment warping limit for the penalized SRVF to 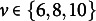 seconds regardless of the true warping limit 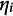 to reflect the real-world cases, where the true warping limit is unknown. Also, to account for individual warping variations, we examined two different settings of 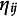 , including a constant 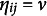 seconds for all 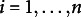 and 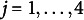 , and a varying 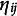 randomly generated from a Gamma distribution 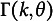 with 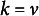 being the shape and 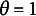 being the scale parameter of the Gamma distribution.

We evaluated the performance of the alignment methods with two metrics. First, we computed the average 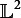 distance between the perceiver’s latent function 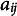 and the estimated functions 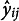 by 
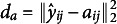
 . The closer 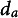 gets to zero, the more accurate estimation of the perceiver’s latent response. Second, we computed the average bias between the true and the estimated correlations to the target, 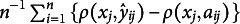 . Here, 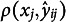 is a commonly used metric for measuring EA, and 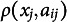 can be considered as the true correlation that the alignment methods aim to achieve. We reported the results for each target separately.

Table 1 shows the performance metrics for the case 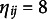 and 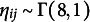 . Results from additional settings, which lead to similar conclusions, are provided in Section S2 of the Supplementary Material. Among the five alignment methods evaluated, the proposed penalized SRVF approach consistently outperforms the others in producing the least biased estimation of EA in all the considered simulation designs. In addition, the average amplitude distance 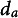 of the proposed penalized SRVF is the smallest for the high negative and high positive targets. For the low negative and low positive targets, the 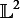 penalized SRVF shows the lowest average 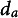 but yields much larger bias. Considering both metrics, the results imply that the proposed method makes the most accurate estimation of the phase shift.

**Table 1:** Performance of different alignment methods in the simulation studies under different warping limits 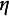 , 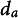 between the estimated perceiver 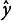 and the true latent perceiver *a*, and the 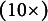 bias of the estimated correlation between the true latent perceiver and the target.

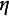	Target	Metric	Pen. SRVF	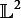 SRVF	Unpen. SRVF	Opt. fixed	No alignment
8	High Neg	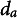	**4.81 (1.36)**	6.74 (3.69)	11.76 (5.1)	6.71 (3.54)	5.62 (1.46)
		Bias	**0.02 (0.39)**	0.67 (0.91)	1.37 (1.53)	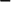 0.36 (1.12)	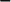 0.17 (0.41)
	Low Neg	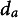	8.33 (5.12)	**4.23 (2.44)**	9.22 (4.04)	9.03 (5.23)	10.56 (6.1)
		Bias	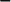 **0.68 (1.79)**	0.69 (1.17)	1.47 (1.75)	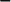 0.82 (2.14)	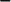 1.55 (2.05)
	High Pos	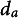	**5.78 (1.62)**	7.17 (3.88)	11.59 (5.24)	7.88 (3.35)	6.04 (1.72)
		Bias	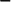 **0.01 (0.87)**	0.63 (1.44)	0.71 (2.25)	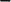 0.21 (1.74)	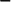 0.13 (0.88)
	Low Pos	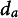	5.92 (1.86)	**4.84 (3.23)**	8.3 (3.52)	7.82 (3.83)	6.59 (1.86)
		Bias	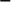 **0.13 (0.61)**	0.55 (0.84)	0.67 (1.1)	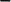 0.76 (1.59)	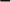 0.31 (0.65)
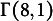	High Neg	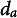	**4.75 (1.82)**	6.64 (3.56)	11.30 (5.03)	6.57 (3.72)	5.52 (1.96)
		Bias	**0.05 (0.43)**	0.72 (0.92)	1.27 (1.44)	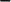 0.28 (1.03)	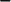 0.14 (0.45)
	Low Pos	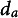	8.02 (4.98)	**4.24 (2.60)**	9.55 (4.24)	9.26 (5.51)	10.49 (5.95)
		Bias	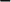 **0.6 (1.73)**	0.68 (1.27)	1.42 (1.78)	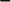 0.86 (2.27)	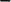 1.46 (1.98)
	High Pos	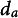	**5.69 (1.93)**	7.16 (3.91)	12.18 (5.81)	7.7 (3.41)	5.94 (2.00)
		Bias	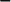 **0.09 (0.83)**	0.59 (1.26)	0.55 (2.52)	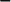 0.32 (1.62)	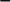 0.20 (0.84)
	Low Pos	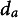	5.98 (2.20)	**4.98 (3.17)**	8.40 (3.53)	7.96 (4.17)	6.57 (2.08)
		Bias	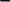 **0.11 (0.73)**	0.68 (0.98)	0.77 (1.31)	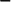 0.66 (1.98)	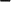 0.29 (0.72)

*Note*: The lowest absolute bias and the lowest 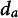 are highlighted for each row. Standard errors are included in the parentheses.

The unpenalized SRVF, optimal fixed, and no alignment methods all yield less accurate estimates of 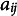 compared to the proposed penalized SRVF. Among them, the unpenalized SRVF produces the largest value of 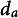 , primarily because it tends to over-align the perceiver’s observed response 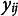 to the target’s rating 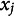 , leading to distorted estimates 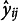 . Additionally, the optimal fixed alignment method often results in the highest standard errors and bias in 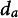 , indicating that it provides inconsistent estimates of the perceiver’s latent ratings.

### 4.2 Simulation 2

In practical applications, the true warping limit 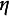 is typically unknown. As a result, the alignment warping limit 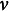 must be chosen based on approximate prior knowledge, which may not perfectly match the true value. To examine the impact of this mismatch, we conducted a simulation study using the same data generation process described in Section 4.1. Specifically, we evaluated 21 different true warping limits 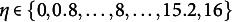 seconds, while fixing the alignment warping limit at 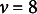 seconds.

**Figure 5:**
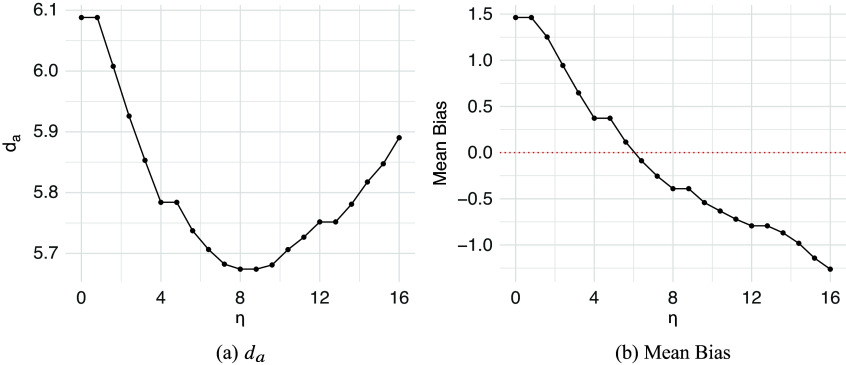
The penalized SRVF alignment results under 21 different true warping limits 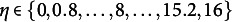 seconds when the upper limit of alignment is 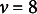 seconds. The red dotted line in the mean bias plot marks the unbiased level.

Figure 5 represents two performance metrics ( 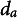 and bias) of the penalized SRVF method across 21 different true warping limits 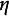 . The results illustrate the method’s robustness to variation in the true warping limit. Notably, when 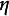 falls within approximately two seconds of the alignment limit 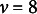 seconds, the penalized SRVF method achieves low 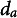 and minimal bias, indicating accurate alignment and estimation. These findings suggest that even without precise knowledge of the true warping limit, selecting 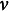 within a reasonable range yields reliable performance, underscoring the method’s practical utility in real-world applications.

### 4.3 Simulation 3

In this simulation, we evaluated the performance of the alignment methods under different levels (i.e., high/medium/low) of EA. Perceivers’ latent ratings 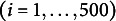 were generated following the idea proposed by Matuk et al. (2022), 
(4.2)

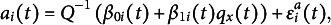

where 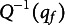 denotes the inverse transformation from an SRVF 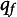 to the original function *f* and 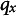 is the SRVF of the target rating for the low intensity positive video *x*. The parameters, 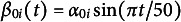 and 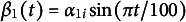 , were chosen based on the setting in Ghosal et al. (2020), with 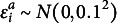 .

We generated perceivers’ observed responses following the same data generation process as in (4.1), setting the alignment warping limit to 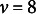 and drawing the true sup-norm limit from a Gamma distribution, 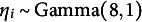 . To simulate varying levels of EA, we defined three conditions: for high EA, 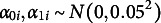 ; for medium EA, 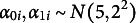 ; and for low EA, 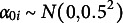 and 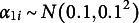 . The resulting average correlations between the target ratings and the perceivers’ latent ratings, 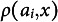 , are approximately 0.81, 0.62, and 0.25 for the high, medium, and low EA conditions, respectively.

Table 2 summarizes two evaluation metrics for the five alignment methods across three levels of EA. The proposed penalized SRVF method demonstrates strong performance regardless of the EA levels. When EA is high, the 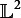 penalized SRVF achieves a slightly lower average 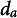 than the penalized SRVF, but the latter yields the lowest average bias. The no alignment method also archives comparable low average bias to the penalized SRVF method. At the medium EA level, the penalized SRVF outperforms all other methods. In the low EA condition, the penalized SRVF’s 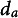 is comparable to the no alignment approach but the no alignment method shows the lowest bias. The benefit of adjusting the observed rating is expected to be limited given the weak association between the target rating and the perceiver’s latent rating. In contrast, the other alignment methods perform significantly worse than the penalized SRVF, particularly under low and medium EA conditions.

**Table 2:** Comparison results based on 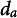 between the estimated perceiver 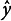 and the true latent perceiver *a* and the 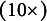 bias of the estimated correlation between the true latent perceiver and the target.

EA	Metric	Pen. SRVF	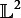 SRVF	Unpen. SRVF	Opt. fixed	No alignment
	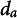	3.33 (0.72)	**3.30 (0.69)**	3.75 (0.58)	4.39 (1.47)	3.57 (0.63)
High	Bias	**0.04 (0.34)**	0.50 (0.29)	0.83 (0.24)	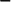 0.50 (0.70)	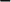 0.08 (0.33)
	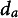	**5.10 (1.75)**	5.77 (3.04)	8.13 (3.11)	6.72 (3.06)	5.33 (1.61)
Med	Bias	**0.01 (0.56)**	0.58 (0.84)	1.43 (0.82)	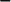 2.17 (2.17)	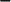 0.22 (0.66)
	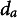	**7.80 (2.62)**	12.08 (5.55)	14.25 (5.07)	11.35 (5.11)	7.82 (2.54)
Low	Bias	0.18 (0.62)	2.06 (1.39)	2.01 (0.87)	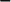 0.11 (2.98)	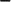 **0.03 (0.65)**

*Note*: The best metrics are highlighted for each row.

## 5 Data application

### 5.1 Study on social empathy

In the first data application, we analyzed a dataset from Devlin et al. (2014), which consists of 121 perceivers’ empathy responses of four distinct videos in which the targets discuss emotional events in their lives. The four videos vary in valence (positive or negative) and intensity (high or low), resulting in four heterogeneous videos, including high-positive, low-positive, high-negative, and low-negative. Participants provided continuous 9-point scale ratings of target emotions while watching each video. These perceiver ratings were compared to the target’s self-ratings. Following standard functional data analysis practices (Srivastava & Klassen, 2016), we preprocessed the data by smoothing target and perceiver ratings using cubic smoothing splines with the default setting of the *smooth.spline* function in R and interpolating the estimated functions on a 300-point equidistant grid within the observed time interval. The goal of the subsequent analysis is to measure the level of EA for each perceiver, quantified by the correlation between the perceiver’s latent ratings and the target’s ratings.

Figure 3 clearly illustrates the misalignment between perceiver and target ratings. Devlin et al. (2014) did not account for this misalignment, measuring EA as a monotonic transformation of the Pearson correlation between the two rating sequences. We applied both unpenalized and penalized SRVF alignments, as these methods offer more flexible time warping than fixed delay alignment. Here, we present results for the penalized alignment with a threshold of 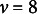 seconds. Results for thresholds of 6 and 10 seconds are included in Section S3.1 of the Supplementary Material.

**Figure 6:**
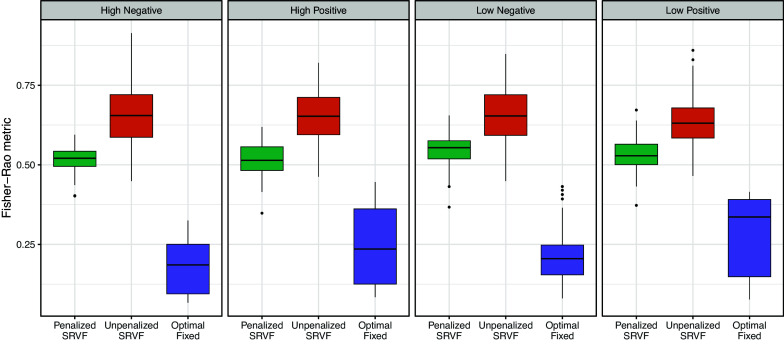
Boxplots for the estimated amount of warping, as measured by the Fisher–Rao metric between the identity warping 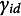 and the estimated warping function using unpenalized SRVF and penalized SRVF method, with 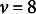 seconds for each video.

To quantify the degree of warping, we computed the phase distance ( 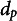 ) between the estimated inverse warping function under each alignment method and the identity warping function for each video. The summary statistics for this measure can be found in Table S2 of the Supplementary Material. Figure 6 reveals that the unpenalized SRVF alignment consistently produces warping functions farther from the identity function than the penalized SRVF alignment, indicating the latter’s effectiveness in reducing excessive warping, where the *p*-values (Table S3 in the Supplementary Material) corresponding to the *t*-tests for the mean differences between penalized SRVF and other method are very close to zero.

We subsequently calculated the Pearson correlation between each perceiver’s aligned ratings and the target’s ratings, which were used as the EA measure. Unlike the simulation studies, the correlation coefficient 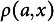 between the target (*x*) and the perceiver’s latent response (*a*) is not observable because the perceiver’s latent response is not known. Instead, we compared these EA measures to those obtained without alignment (identity warping), referred to as identity correlations. Notably, about 2% of the cases exhibited negative correlations between perceiver and target ratings under identity warping. As a data pre-processing step, we removed those cases based on the concern that they may exhibit fundamentally different empathy patterns from the general population of perceivers.

**Figure 7:**
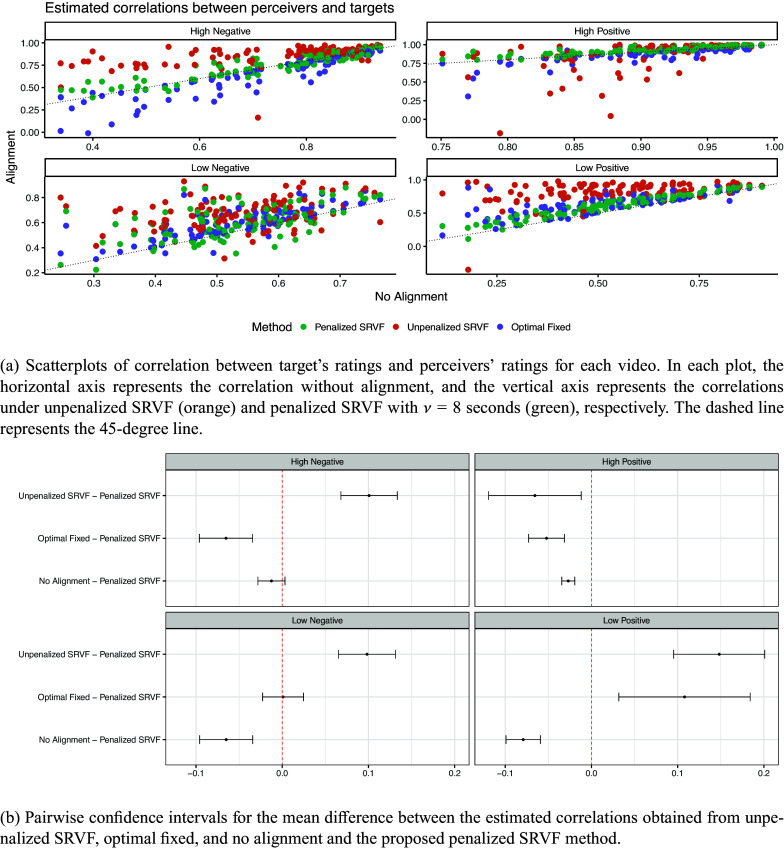
Results for EA estimates in the social EA study.

Figure 7a presents scatterplots comparing these EA measures between target and perceiver ratings for pre- and post-aligned data across the four video conditions. The majority of points reside above the 45-degree line, indicating that accounting for misalignment generally increases EA measures compared to unaligned analyses. However, the unpenalized SRVF alignment often inflates EA considerably, as observed in the simulation results This is most pronounced in the low intensity positive video group (bottom right panel of Figure 7a), where many unpenalized EA approach one, implying near-perfect empathy for most perceivers, an unrealistic outcome given the video’s low expressiveness. Conversely, for the high intensity positive video group (top right panel), some unpenalized EA fell substantially below identity EA because excessive warping distorted overall function trends.

The proposed penalized SRVF alignment provides a reasonable compromise between the identity EA and the unpenalized SRVF EA. For low-expressivity videos (bottom row, Figure 7a), penalized alignment EA measures generally exceed those from no alignment, likely due to increased misalignment challenges under reduced emotional cues. It is also interesting to find that for videos under positive emotion (second column, Figure 7a), the EA with the penalized SRVF alignment has a trivial difference compared with the EA with no alignment, while for videos under negative emotion (first column, Figure 7a), the difference is much bigger. This suggests a stronger time-warping effect for negative emotions, which is consistent with psychological research indicating better recognition of positive emotions (Bandyopadhyay et al., 2021). Figure 7b shows that the penalized SRVF methods lead to estimated EA with a significantly smaller mean than those obtained from unpenalized SRVF in all four videos. In three out of four videos, the mean EA obtained from the penalized SRVF is also significantly larger than those from no alignment, and generally differs significantly from those obtained under the optimal fixed delay method. In addition, Figure S1 in the Supplementary Material shows the correlations among the EA measures obtained by different alignment methods. Although they are positively correlated with others, they are not equivalent and our proposed alignment method can lead to improved inference in a downstream analysis.

We also examined the associations between perceiver-specific trait positive emotion and their EA. Trait positive emotion reflects a perceiver’s stable tendency to experience positive emotions across diverse situations and over time, and it is typically associated with greater sociability, prosocial behavior, and openness (Devlin et al., 2014). To make this analysis consistent with the approach adopted by Devlin et al. (2014), we fitted a simple linear regression model with the Fisher transformed EA measure as the outcome and trait positive emotion as the predictor. Table 3 summarizes the estimated slope of each regression. It reveals that the penalized SRVF method consistently yields larger absolute coefficient estimates than the no-alignment approach across all four videos. This pattern is not consistently observed with the unpenalized SRVF or the fixed delay methods. These findings suggest that failing to properly address misalignment may obscure important relationships between EA and perceiver characteristics.

**Table 3:** Estimated coefficients for Trait Positive Affect as a predictor of EA as measured by different alignment methods.

	High negative	Low negative	High positive	Low positive
No alignment	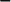 0.013 (0.005)*	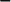 0.003 (0.002)	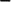 0.011 (0.006)	0.012 (0.005)*
Fixed delay	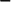 0.017 (0.006)*	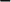 0.004 (0.003)	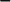 0.017 (0.007)*	0.005 (0.004)
Unpenalized SRVF	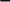 0.009 (0.005)*	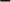 0.002 (0.003)	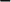 0.024 (0.011)*	0.019 (0.009)*
Penalized SRVF (8s)	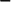 0.014 (0.005)*	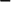 0.004 (0.003)	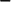 0.016 (0.008)*	0.013 (0.005)*

*Note*: Standard errors are included in parentheses and * indicates significance at the significance level 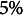 .

### 5.2 Study on music empathy

Tabak et al. (2022) conducted an EA study investigating three primary emotions: joy/happiness, sadness, and anger ( 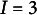 ). For each emotion, three original solo piano pieces ( 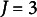 ) were composed and performed by experienced musicians. These pieces were designed to evoke the target emotions within familiar musical styles (classical, popular, and jazz). Both musicians (as targets) and 123 participants (as perceivers) rated the emotional content of each piece on a 9-point scale. As with the previous dataset, we preprocessed the data by smoothing and interpolating rating functions.

Unlike the correlation-based approach, Tabak et al. (2022) employed a linear mixed-effect model for a more nuanced analysis of EA. This model decomposed perceiver responses into three latent factors: bias, discrimination, and variance. Bias represented the systematic deviation between perceiver and target ratings, while discrimination captured a perceiver’s sensitivity to changes in the target’s expressed emotion. Finally, variance accounted for random noise in perceiver ratings.

Within each group of emotion ( 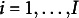 ), let 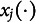 and 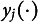 be the target and a perceiver’s ratings, respectively, for the *j*th stimulus. Tabak et al. (2022) proposed the following linear mixed-effect model to describe the relation between 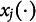 and 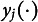 : 
(5.1)

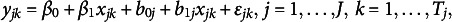

where 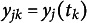 and 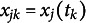 are the perceiver and target’s respective ratings at the *k*th time point, and 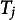 is the number of points for the *j*th stimuli. The (fixed) intercept 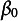 and slope 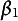 represent a perceiver’s mean bias and discrimination ability across all the *J* stimuli, respectively. The random intercept 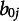 , random slope 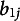 , and the random noise 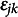 are assumed to follow a normal distribution with zero mean and respective variance component 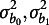 , and 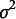 , which represents the variability of bias, discrimination, and random noise across different stimuli. This model treats ratings as discrete points and does not account for potential misalignments between perceiver and target responses.

To address this limitation, we integrated an alignment step into the model framework. Treating the observed ratings as sampled points from corresponding functions, we applied and compared penalized and unpenalized time-warping SRVF alignments to account for potential misalignments. Let 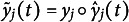 be the estimated aligned function with 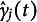 being an estimated inverse warping function from aligning 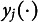 with 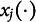 , we then modeled 
(5.2)

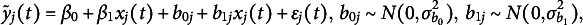

where 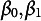 , 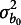 , 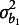 , and 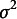 in Model (5.2) maintain the same interpretations as in Model (5.1).

We fitted Model (5.2) for each perceiver and primary emotion. Using the lme4 package in R (Bates et al., 2015), we employed restricted maximum likelihood estimation to obtain parameter estimates 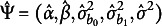 and best linear unbiased predictions (BLUPs) of random effects 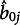 and 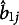 for 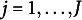 . To assess the impact of time warping, we compared parameter estimates 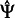 under no alignment (i.e., 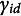 ), the unpenalized SRVF ( 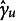 ), and the penalized SRVF alignment ( 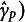 , setting the penalty threshold at 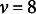 seconds for the penalized alignment. Results for 6- and 10-second thresholds are provided in Section S3.2 of the Supplementary Material.

To assess model fit, we computed two metrics: average warping and average goodness of fit across all *J* tasks. The first metric, average Fisher–Rao distance, quantifies the mean warping magnitude relative to the identity warping: 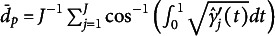 . A higher 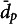 indicates greater warping. The second metric measures the vertical distance between the estimated aligned response function and the fitted value function. Specifically, letting 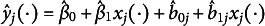 , this vertical distance is calculated to be the 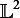 distance between the estimated aligned response 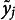 and the fitted value function 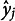 , i.e., 
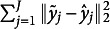
 , where a lower value signifies a better model fit.

**Figure 8:**
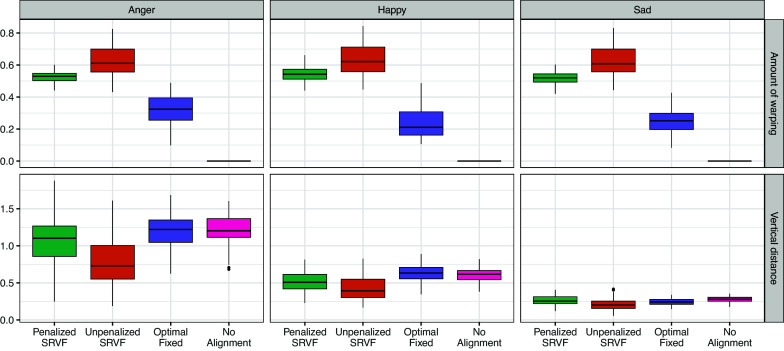
Boxplots of the metrics for the average amount of warping (top row) and goodness of fit (bottom row) of the estimated models for the three sets of music recordings. The penalized SRVF alignment was conducted using the threshold 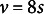 .

Figure 8 reveals that the no alignment model exhibits significantly inferior fit compared to the other two approaches (all the *p*-values for comparing pairwise mean differences are close to zero, see Table S4 in the Supplementary Material). This underscores the importance of addressing misalignment between perceiver and target ratings to prevent model underfitting. Similar to our simulation findings, the unpenalized SRVF alignment demonstrates overfitting, sacrificing model fit for excessive warping. In contrast, the penalized alignment method provides a reasonable compromise between these extremes, enhancing model fit while mitigating overfitting through judicious penalty application.

**Figure 9:**
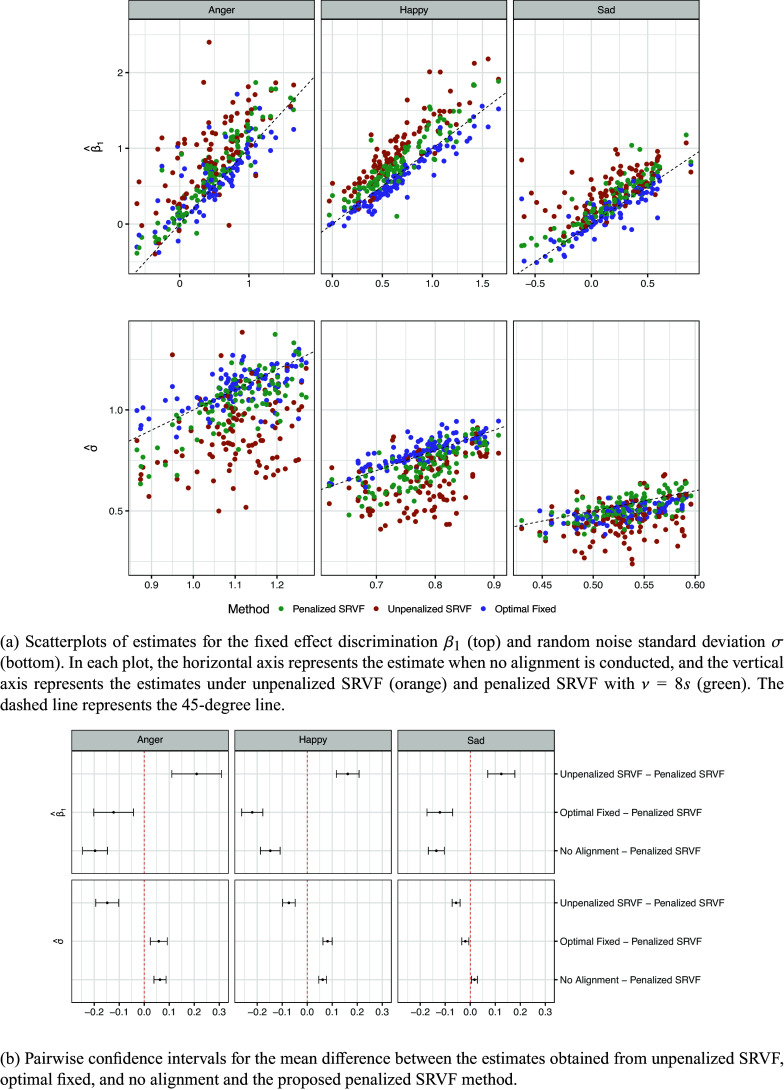
Results for parameter estimates in the music EA study.

Figure 9a compares parameter estimates (aligned versus unaligned) for the fixed effect discrimination ( 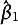 ) and random noise standard deviation ( 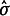 ) across the three emotion groups, and Figure 9b shows the pairwise confidence intervals in the mean estimates of the penalized SRVF method against other alignment methods. In the top row of both figures, while both unpenalized SRVF and penalized SRVF alignment methods tend to increase the discrimination estimates 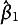 , the optimal fixed delay tends to decrease it compared to the no alignment. However, similar to the social empathy study in Section 5.1, the unpenalized SRVF alignment increases this discrimination estimate much more than the penalized SRVF, making the unpenalized SRVF more prone to overfit. This conclusion is further evidenced in the bottom row, where the estimated standard deviation 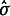 from the unpenalized SRVF method is substantially smaller than that from the other two alignment methods.

## 6 Discussion

In emotional perception research, misalignment caused by complex cognitive decoding processes and the time needed to enact a behavioral response is a well-established phenomenon. In EA studies, the discrepancy between the perceiver’s observed rating and the target’s rating is influenced by both the misalignment not due to lack of EA and the psychologically meaningful disagreement resulting from lack of EA. Yet, most of the conventional EA studies either ignore this kind of misalignment or apply an oversimplified fixed delay for adjustment, where both options can lead to biased results. This study introduces a novel, flexible approach using a new constrained optimization problem based on the SRVF representation of the functions to reduce the misalignment, which varies from individual to individual. Considering realistic conditions of the warping process, our simulation studies demonstrate that the proposed penalized SRVF alignment method provides improved estimates of the true EA measure compared to existing approaches. In two case studies on social and music empathy, this method yields plausible EA measures, which subsequently reveals more potential associations between EA and perceivers’ characteristics.

The proposed penalized alignment approach offers several advantages. 1) Individualized adjustments: It tailors alignment to unique patterns of misalignment for each perceiver. 2) Prevention of over-alignment: It incorporates a natural constraint on the extent of allowable warping. 3) Simplicity and interpretability: The penalty term can be easily set up by using the maximum delay in perceivers’ responses, where it is straightforward to use empirical evidence and expert opinion. Moreover, EA studies often vary in context and focus. In situations, where reaction time is less critical, such as listening to a friend’s long story, a broader penalization window may be appropriate. Conversely, in high-stakes scenarios like heated arguments, a narrower window can better reflect the urgency of responses. This flexibility enables researchers to tailor penalization parameters to the specific demands of each study. By integrating these features, our approach enhances the accuracy of downstream EA analyses, including correlational studies and complex linear mixed models.

The core component in our proposed method, the warping functions, has been widely employed to correct misalignment in fields like physics and biology, where objective benchmarks exist. To the best of our knowledge, the application of warping functions to the abstract and subjective domain of human perception is unexplored. In this study, we have demonstrated their effectiveness and flexibility in adjusting individually varying misalignment across different types of emotional stimuli (visual and audio). It further expands the potential for using warping functions in new research areas.

Future research could focus on several key areas. One potential direction is to model the similarity of warping functions of the same individual across different stimuli by introducing random effects. Another area of interest could be to develop a new EA alignment method by incorporating additional data, such as the functional magnetic resonance imaging (fMRI) blood-oxygen-level-dependent (BOLD) signals of targets and perceivers during rating assessments, which could help detect true emotional changes. Although our method accurately identified simulated noise and showed favorable psychometric characteristics, we caution against interpreting the corrected scores from our method as definitive indicators of EA devoid of all measurement error. Future work is needed to explicitly test the extent to which the penalized alignment approach can distinguish measurement noise from meaningful differences in EA, for example, by experimentally manipulating whether participants can pause the video to make ratings or by varying the cognitive load placed on participants. Finally, we note that these analyses were exploratory in nature. Given the methodological flexibility of the proposed method and the number of analytic decisions involved (e.g., the upper limit of warping functions), future work can aim to replicate these findings using pre-registered designs to increase confidence in the robustness of the results provided by the penalized SRVF alignment method.

## Supporting information

Nghiem et al. supplementary material Nghiem et al. supplementary material

## Data Availability

The data used in the two case studies, along with the R codes that implement the penalized SRVF alignment method and produce numerical results in the article, are available at the GitHub repository: https://github.com/chulmoon/EA_Alignment.

## Supplementary material

The supplementary material for this article can be found at https://doi.org/10.1017/psy.2025.10040.
