# Supplementary material for: Enhancing Empathic Accuracy: Penalized Functional Alignment Method to Correct Temporal Misalignment in Real-Time Emotional Perception
Source: Psychometrika. 2025 Sep 5;90(4):1536–57. doi: 10.1017/psy.2025.10040 (PMC12660001; doi:10.1017/psy.2025.10040)
Supplement: Nghiem et al. supplementary material [file S0033312325100409sup001.pdf]

Supplementary Material for "Enhancing Empathic Accuracy: Penalized Functional Alignment Method to Correct Temporal Misalignment in Real-time Emotional Perception"

S1 Proof of Lemma 3.1

Let Γ_I^* be the set of warping functions under penalized warping of (3.2) in the paper, $\Gamma_I^* = \{\gamma : [0,1] \to [0,1]$, $\gamma(0) = 0$, $\gamma(1) = 1$, γ is invertible, γ and γ^{-1} are smooth, $\sup |\gamma - \gamma_{id}| \leq \nu\}$. By representing $\gamma \in \Gamma_I^*$ as its SRVF $\sqrt{\gamma'}$, the space Γ_I^* maps to $\mathcal{Q}^* = \{\sqrt{\gamma'} : [0,1] \mapsto \mathbb{R}_{\geq 0} \mid \int_0^1 \left(\sqrt{\gamma'(s)}\right)^2 dt = 1, \left(\int_0^t \left(\sqrt{\gamma'(s)}\right)^2 ds - t\right)^2 \leq \nu^2$ for $0 \leq t \leq 1\}$. Here, $\Gamma_I^* \subset \Gamma_I$ and $\mathcal{Q}^* \subset \mathcal{Q} = \{\sqrt{\dot{\gamma}} : [0,1] \mapsto \mathbb{R}_{\geq 0} \mid \int_0^1 \left(\sqrt{\dot{\gamma}(s)}\right)^2 dt = 1\}$. Because $\mathcal{Q}$ is a subset of the positive orthant of the unit Hilbert sphere $\mathbb{S}_\infty^+$, $\mathcal{Q}^*$ also is a subset of $\mathbb{S}_\infty^+$. Therefore, the SRVF of warping functions from the penalized SRVF are the elements of a unit Hilbert sphere, and their distance d_p is the arc-length distance. It can be approximated by $d_p(x,y) \approx \cos^{-1}\left(\int_0^1 \sqrt{\hat{\gamma}_p'(t)} dt\right)$.

S2 Supplements to Simulation Study

Table S1: Performance of different alignment methods in the simulation studies under different warping limits η, measured by the $\mathbb{L}^2$ distance d_a between the aligned perceiver $\hat{y}$ and the true latent perceiver a, and the (10×) bias of the estimated correlation between the true latent perceiver and the target. The lowest absolute bias and the lowest d_a are highlighted for each row. Standard errors are included in the parentheses.

η	Video	Metric	Pen. SRVF	$\mathbb{L}^2$ SRVF	Unpen. SRVF	Opt. Fixed	No Alignment
6	High Neg	d_a	**3.74 (1.02)**	6.63 (3.58)	11.72 (5.13)	4.64 (1.81)	4.47 (1.27)
		Bias	**0.03 (0.28)**	0.67 (1.00)	1.22 (1.41)	-0.10 (0.51)	-0.09 (0.31)
	Low Neg	d_a	6.95 (4.38)	**3.80 (2.35)**	9.01 (4.13)	8.00 (5.22)	8.96 (5.36)
		Bias	-0.63 (1.43)	**0.61 (1.10)**	1.35 (1.70)	-0.80 (2.09)	-1.30 (1.62)
	High Pos	d_a	**4.47 (1.20)**	6.65 (3.87)	11.83 (5.53)	6.112 (2.94)	4.69 (1.27)
		Bias	**-0.00 (0.59)**	0.67 (1.24)	0.80 (2.27)	-0.08 (1.22)	-0.08 (0.61)
	High Neg	d_a	**4.48 (1.35)**	4.52 (3.03)	7.94 (3.48)	6.00 (3.20)	5.07 (1.40)
		Bias	**-0.06 (0.47)**	0.60 (0.85)	0.74 (1.20)	-0.41 (1.21)	-0.20 (0.49)
$\Gamma(6,1)$	High Neg	d_a	**3.88 (1.71)**	6.37 (3.45)	11.25 (4.84)	4.72 (2.41)	4.49 (1.85)
		Bias	**0.04 (0.31)**	0.60 (0.91)	1.18 (1.35)	-0.16 (0.56)	-0.08 (0.33)
	Low Neg	d_a	6.70 (4.51)	**4.05 (2.68)**	9.27 (4.09)	7.79 (4.84)	8.74 (5.44)
		Bias	**-0.52 (1.46)**	0.66 (1.22)	1.43 (1.71)	-0.63 (1.95)	-1.14 (1.66)
	High Pos	d_a	**4.50 (1.54)**	6.67 (3.87)	12.38 (5.97)	6.21 (2.98)	4.70 (1.63)
		Bias	**-0.04 (0.59)**	0.59 (1.22)	0.61 (2.43)	-0.14 (1.24)	-0.12 (0.60)
	High Neg	d_a	4.54 (1.70)	**4.41 (2.76)**	8.33 (3.37)	5.81 (3.37)	5.16 (1.73)
		Bias	**-0.07 (0.50)**	0.59 (0.90)	0.71 (1.29)	-0.27 (1.20)	-0.22 (0.52)
10	High Neg	d_a	**5.94 (1.78)**	6.63 (3.58)	11.58 (5.30)	8.36 (4.21)	6.88 (1.88)
		Bias	**0.01 (0.53)**	0.67 (1.00)	1.23 (1.43)	-0.52 (1.27)	-0.23 (0.52)
	Low Neg	d_a	9.80 (5.83)	**3.80 (2.35)**	9.20 (4.06)	10.67 (5.54)	12.47 (6.72)
		Bias	-0.87 (2.07)	**0.61 (1.10)**	1.43 (1.83)	-1.14 (2.57)	-1.98 (2.35)
	High Pos	d_a	6.92 (1.94)	**6.65 (3.87)**	11.99 (5.17)	9.31 (3.88)	7.25 (2.03)
		Bias	**-0.07 (1.00)**	0.67 (1.24)	0.70 (2.56)	-0.49 (2.15)	-0.22 (1.02)
	High Neg	d_a	7.12 (2.32)	**4.52 (3.03)**	8.17 (3.24)	9.48 (4.77)	7.73 (2.18)
		Bias	**-0.18 (0.80)**	0.60 (0.85)	0.69 (1.12)	-0.90 (2.31)	-0.39 (0.81)
$\Gamma(10,1)$	High Neg	d_a	**5.69 (2.12)**	6.37 (3.45)	11.25 (4.69)	7.78 (3.95)	6.62 (2.25)
		Bias	**0.04 (0.50)**	0.60 (0.91)	1.20 (1.34)	-0.42 (1.13)	-0.19 (0.51)
	Low Neg	d_a	**9.27 (5.51)**	4.05 (2.68)	9.07 (4.08)	10.57 (5.84)	11.97 (6.54)
		Bias	**-0.77 (2.01)**	0.66 (1.22)	1.34 (1.61)	-1.21 (2.67)	-1.88 (2.33)
	High Pos	d_a	6.76 (2.17)	**6.67 (3.87)**	11.68 (5.72)	8.97 (3.75)	7.10 (2.27)
		Bias	**-0.05 (1.00)**	0.59 (1.22)	0.79 (2.25)	-0.45 (2.05)	-0.21 (1.02)
	High Neg	d_a	7.00 (2.48)	**4.41 (2.76)**	8.34 (3.61)	9.16 (4.68)	7.66 (2.43)
		Bias	**-0.10 (0.88)**	0.59 (0.90)	0.63 (1.22)	-0.94 (2.28)	-0.32 (0.89)

S3 Supplementary Results for Data Applications

S3.1 Data application: Social empathy

This section contains additional results for the first data application, including the estimates of the amount of warping and correlation between the aligned perceivers and the targets under different thresholds.

Table S2: Mean (standard deviation) of the estimated amount of warping and correlation between aligned perceiver and the target across 122 perceivers in the social empathy study, under different alignment methods, including no alignment, unpenalized SRVF, and penalized SRVF with thresholds $\nu \in \{6, 8, 10\}$ seconds.

Video	Metric	No alignment	Unpen. SRVF	Pen. SRVF		
				$\nu = 6$	$\nu = 8$	$\nu = 10$
High Negative	Correlation	0.51 (0.14)	0.68 (0.11)	0.58 (0.15)	0.59 (0.16)	0.58 (0.17)
	Amount of Warping	0.00 (0.00)	0.66 (0.09)	0.50 (0.05)	0.55 (0.05)	0.57 (0.05)
High Positive	Correlation	0.73 (0.20)	0.85 (0.13)	0.75 (0.19)	0.75 (0.19)	0.75 (0.19)
	Amount of Warping	0.00 (0.00)	0.66 (0.10)	0.50 (0.03)	0.52 (0.04)	0.53 (0.04)
Low Negative	Correlation	0.89 (0.15)	0.85 (0.26)	0.91 (0.15)	0.91 (0.15)	0.91 (0.15)
	Amount of Warping	0.00 (0.00)	0.65 (0.08)	0.49 (0.05)	0.52 (0.05)	0.53 (0.05)
Low Positive	Correlation	0.46 (0.28)	0.68 (0.44)	0.50 (0.29)	0.52 (0.30)	0.54 (0.31)
	Amount of Warping	0.00 (0.00)	0.64 (0.07)	0.49 (0.04)	0.53 (0.05)	0.54 (0.05)

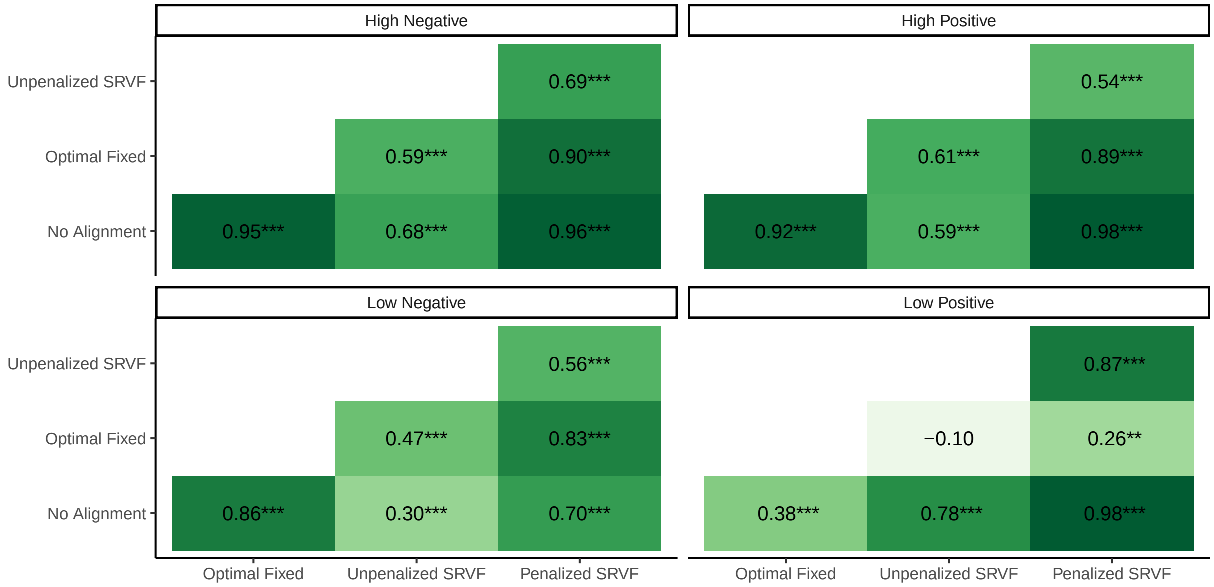

Figure S1: Correlations among EA measures obtained by different alignment methods in the social EA study

Table S3: Point estimates, 95% confidence intervals and p-values for the difference in the estimated amount of warping obtained by the proposed penalized SRVF with warping limit $\nu = 8s$ versus that obtained by the other alignment method.

Video	Pair	Est	Low CI	Upp CI	p-value
High Negative	No Alignment - Penalized SRVF	-0.52	-0.53	-0.51	0.00
	Optimal Fixed - Penalized SRVF	-0.33	-0.35	-0.31	0.00
	Unpenalized SRVF - Penalized SRVF	0.14	0.12	0.16	0.00
High Positive	No Alignment - Penalized SRVF	-0.55	-0.56	-0.53	0.00
	Optimal Fixed - Penalized SRVF	-0.33	-0.36	-0.31	0.00
	Unpenalized SRVF - Penalized SRVF	0.11	0.08	0.13	0.00
Low Negative	No Alignment - Penalized SRVF	-0.52	-0.53	-0.50	0.00
	Optimal Fixed - Penalized SRVF	-0.26	-0.30	-0.23	0.00
	Unpenalized SRVF - Penalized SRVF	0.13	0.11	0.15	0.00
Low Positive	No Alignment - Penalized SRVF	-0.53	-0.54	-0.52	0.00
	Optimal Fixed - Penalized SRVF	-0.26	-0.29	-0.22	0.00
	Unpenalized SRVF - Penalized SRVF	0.10	0.08	0.13	0.00

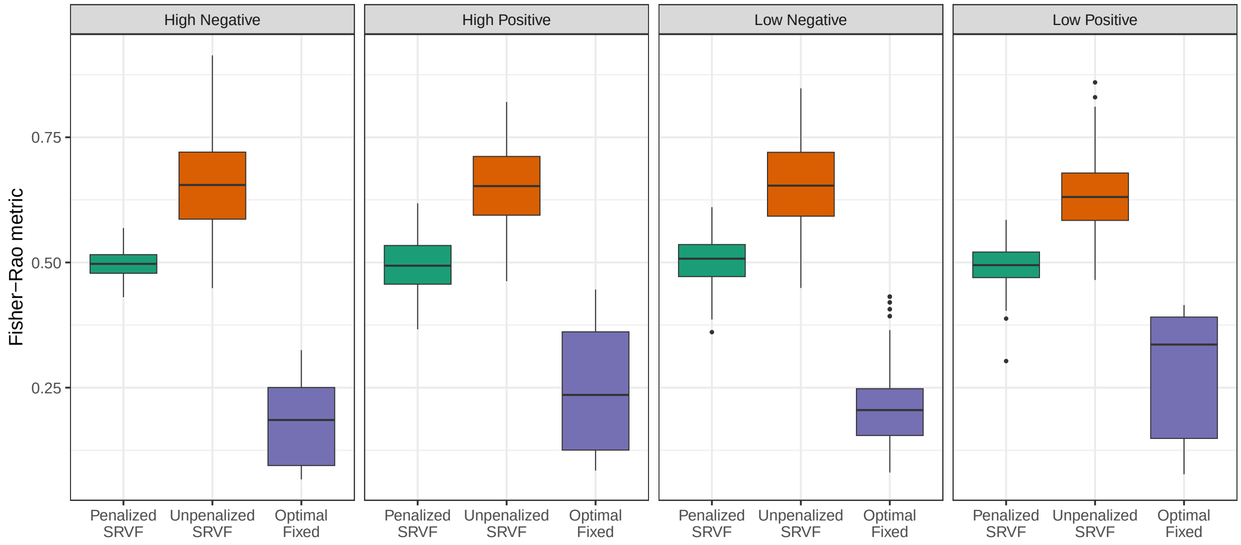

Figure S2: Boxplots for the estimated amount of warping, as measured by the Fisher-Rao metric between the identity warping γ_{id} and the estimated warping function using unpenalized SRVF and penalized SRVF method with $\nu = 6s$ for each video.

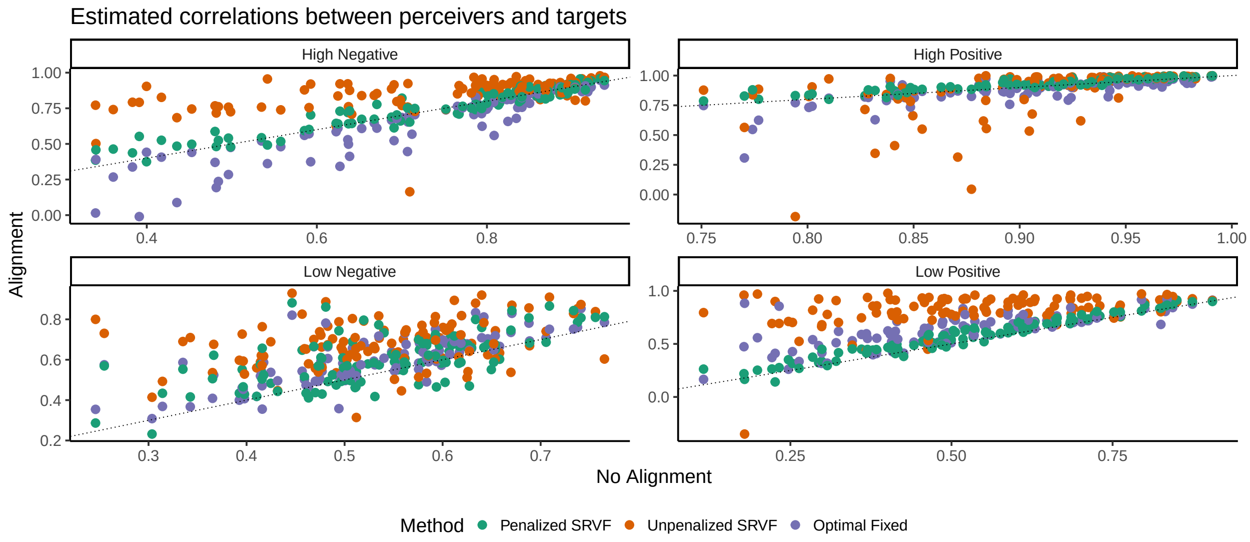

Figure S3: Scatterplots of correlation between target's ratings and each perceiver's ratings for each video. In each plot, the horizontal axis represents the correlation when no alignment is conducted, and the vertical axis represents the estimates under unpenalized SRVF (orange) and penalized SRVF with $\nu = 6s$ (green).

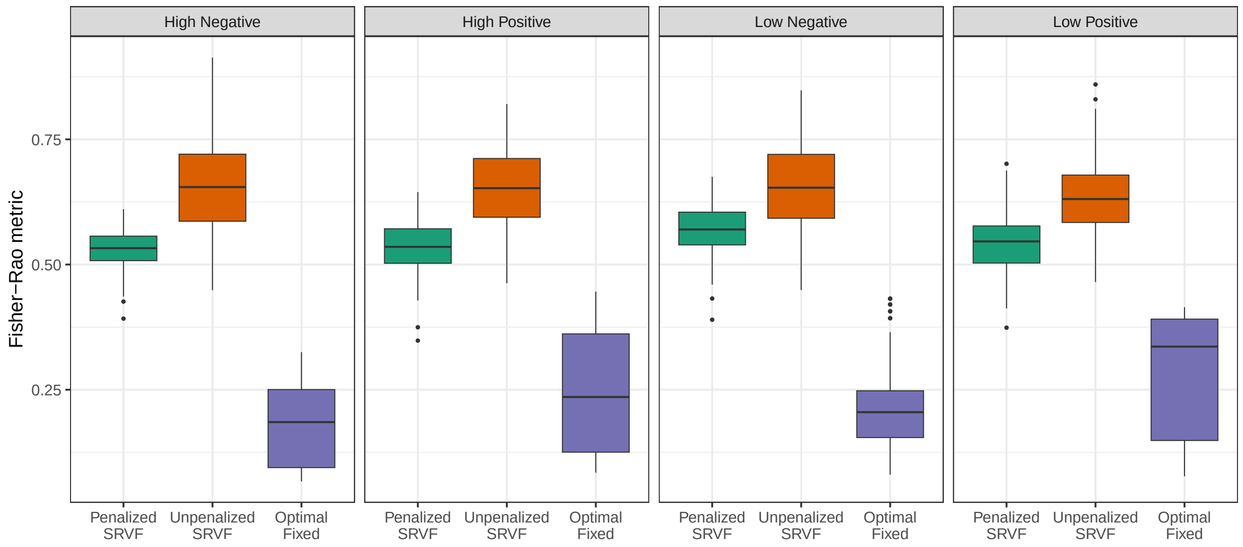

Figure S4: Boxplots for the estimated amount of warping, as measured by the Fisher-Rao metric between the identity warping γ_{id} and the estimated warping function using unpenalized SRVF and penalized SRVF method with $\nu = 10s$ for each video.

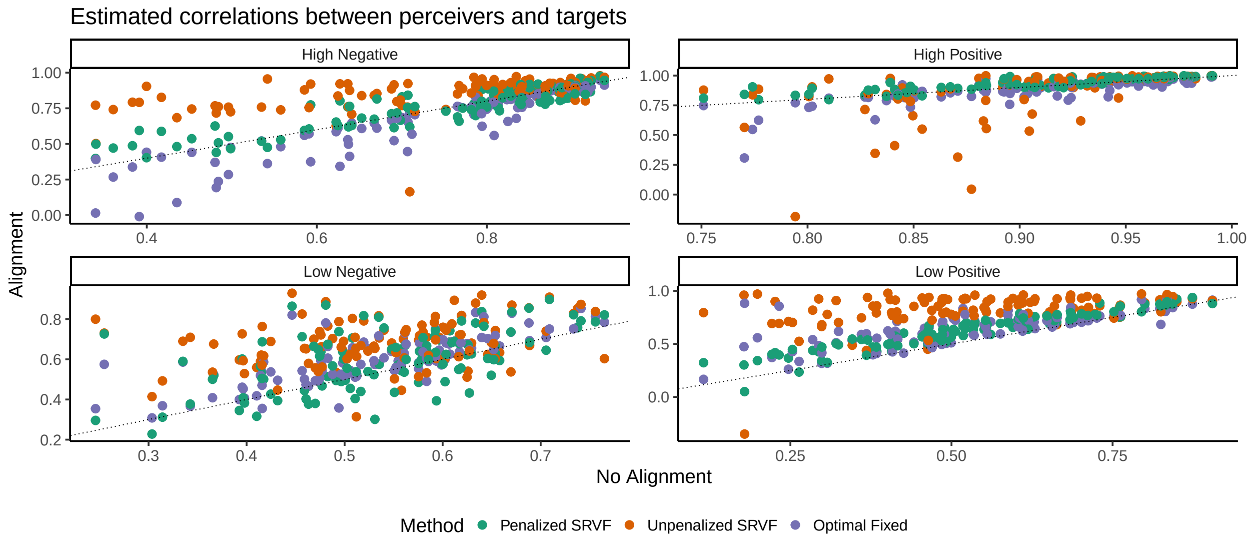

Figure S5: Scatterplots of correlation between target's ratings and each perceiver's ratings for each video. In each plot, the horizontal axis represents the correlation when no alignment is conducted, and the vertical axis represents the estimates under unpenalized SRVF (orange) and penalized SRVF with $\nu = 10s$ (green).

S3.2 Data application: Music empathy

This section contains additional results for the first data application, including the estimates of the amount of warping and all parameters under different thresholds.

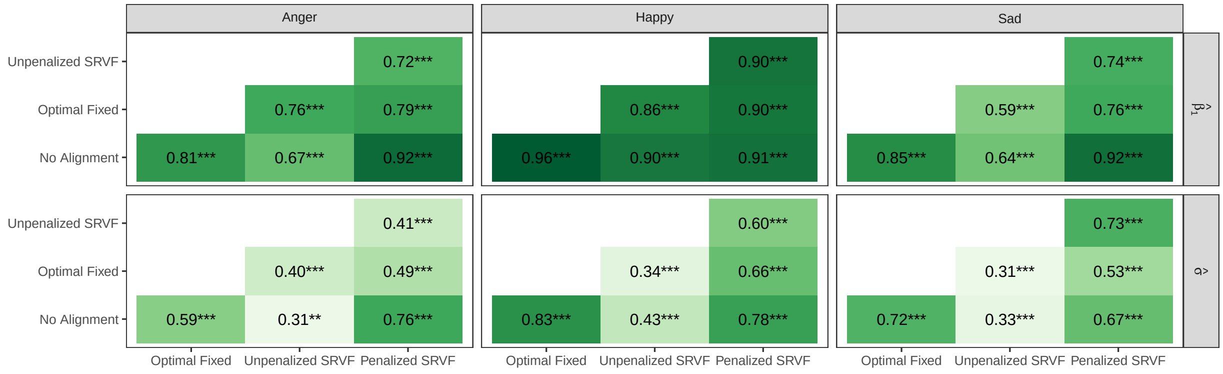

Figure S6: Correlation among EA measures (discrimination, $\hat{\beta}_1$ and standard deviation of random noise, $\hat{\sigma}$) in music EA study obtained by different alignment methods, including no alignment, optimal fixed delay, unpenalized SRVF, and penalisczed SRVF with $\nu = 8s$

.

Table S4: Pairwise confidence intervals and p-values for the mean difference in the vertical distance and amount of warping obtained from different alignment methods in the music EA study, including no alignment, optimal fixed delay, unpenalized SRVF, and penaliszed SRVF with $\nu = 8s$.

Music	Metric	Pair	Est	Low CI	Upp CI	p-value
Anger	Amount of Warping	Penalized SRVF - Unpenalized SRVF	-0.095	-0.114	-0.076	0.000
		Penalized SRVF - Optimal Fixed	0.312	0.289	0.334	0.000
		Penalized SRVF - No Alignment	0.545	0.534	0.557	0.000
		Unpenalized SRVF - Optimal Fixed	0.412	0.382	0.443	0.000
		Unpenalized SRVF - No Alignment	0.640	0.615	0.666	0.000
		Optimal Fixed - No Alignment	0.238	0.213	0.263	0.000
	Vertical distance	Penalized SRVF - Unpenalized SRVF	0.094	0.061	0.127	0.000
		Penalized SRVF - Optimal Fixed	-0.118	-0.144	-0.091	0.000
		Penalized SRVF - No Alignment	-0.087	-0.108	-0.065	0.000
		Unpenalized SRVF - Optimal Fixed	-0.212	-0.251	-0.173	0.000
		Unpenalized SRVF - No Alignment	-0.182	-0.218	-0.147	0.000
		Optimal Fixed - No Alignment	0.031	0.016	0.047	0.000
Happy	Amount of Warping	Penalized SRVF - Unpenalized SRVF	-0.099	-0.117	-0.080	0.000
		Penalized SRVF - Optimal Fixed	0.209	0.186	0.232	0.000
		Penalized SRVF - No Alignment	0.526	0.518	0.535	0.000
		Unpenalized SRVF - Optimal Fixed	0.310	0.283	0.337	0.000
		Unpenalized SRVF - No Alignment	0.626	0.604	0.648	0.000
		Optimal Fixed - No Alignment	0.320	0.295	0.345	0.000
	Vertical distance	Penalized SRVF - Unpenalized SRVF	0.277	0.192	0.362	0.000
		Penalized SRVF - Optimal Fixed	-0.117	-0.186	-0.047	0.000
		Penalized SRVF - No Alignment	-0.128	-0.179	-0.077	0.000
		Unpenalized SRVF - Optimal Fixed	-0.389	-0.467	-0.311	0.000
		Unpenalized SRVF - No Alignment	-0.410	-0.487	-0.332	0.000
		Optimal Fixed - No Alignment	-0.019	-0.067	0.029	0.290
Sad	Amount of Warping	Penalized SRVF - Unpenalized SRVF	-0.106	-0.127	-0.086	0.000
		Penalized SRVF - Optimal Fixed	0.271	0.251	0.290	0.000
		Penalized SRVF - No Alignment	0.518	0.509	0.528	0.000
		Unpenalized SRVF - Optimal Fixed	0.378	0.353	0.403	0.000
		Unpenalized SRVF - No Alignment	0.625	0.601	0.649	0.000
		Optimal Fixed - No Alignment	0.250	0.230	0.270	0.000
	Vertical distance	Penalized SRVF - Unpenalized SRVF	0.052	0.039	0.066	0.000
		Penalized SRVF - Optimal Fixed	0.021	0.007	0.036	0.000
		Penalized SRVF - No Alignment	-0.015	-0.027	-0.003	0.001
		Unpenalized SRVF - Optimal Fixed	-0.032	-0.052	-0.012	0.000
		Unpenalized SRVF - No Alignment	-0.069	-0.088	-0.050	0.000
		Optimal Fixed - No Alignment	-0.037	-0.046	-0.029	0.000

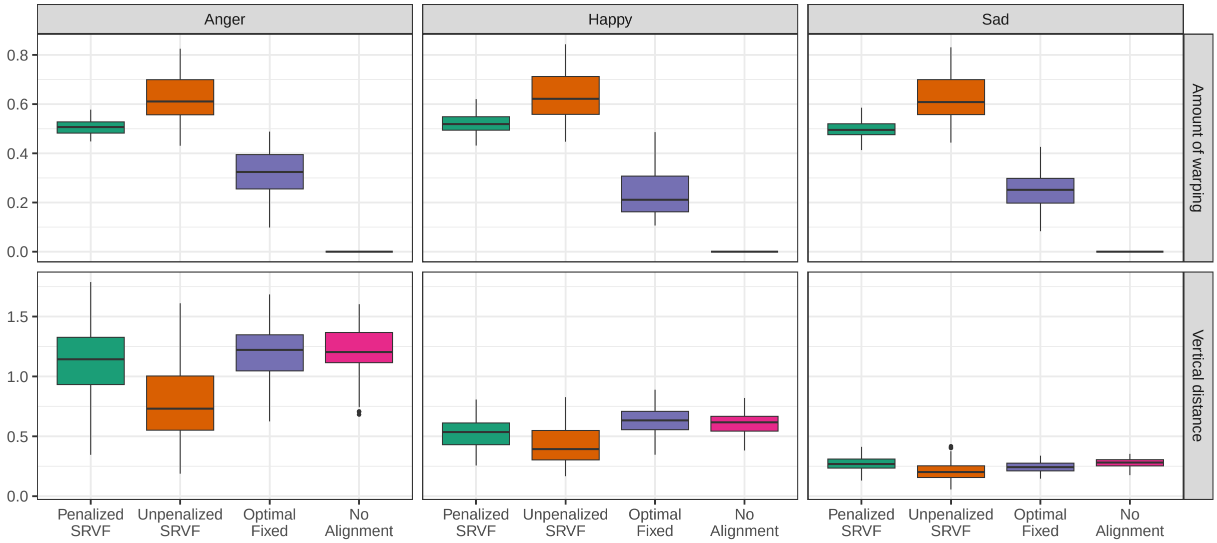

Figure S7: Boxplots of the vertical distance and the average amount of warpings of the estimated models for the three sets of music recordings. The penalized SRVF alignment was conducted using the threshold $\nu = 6s$.

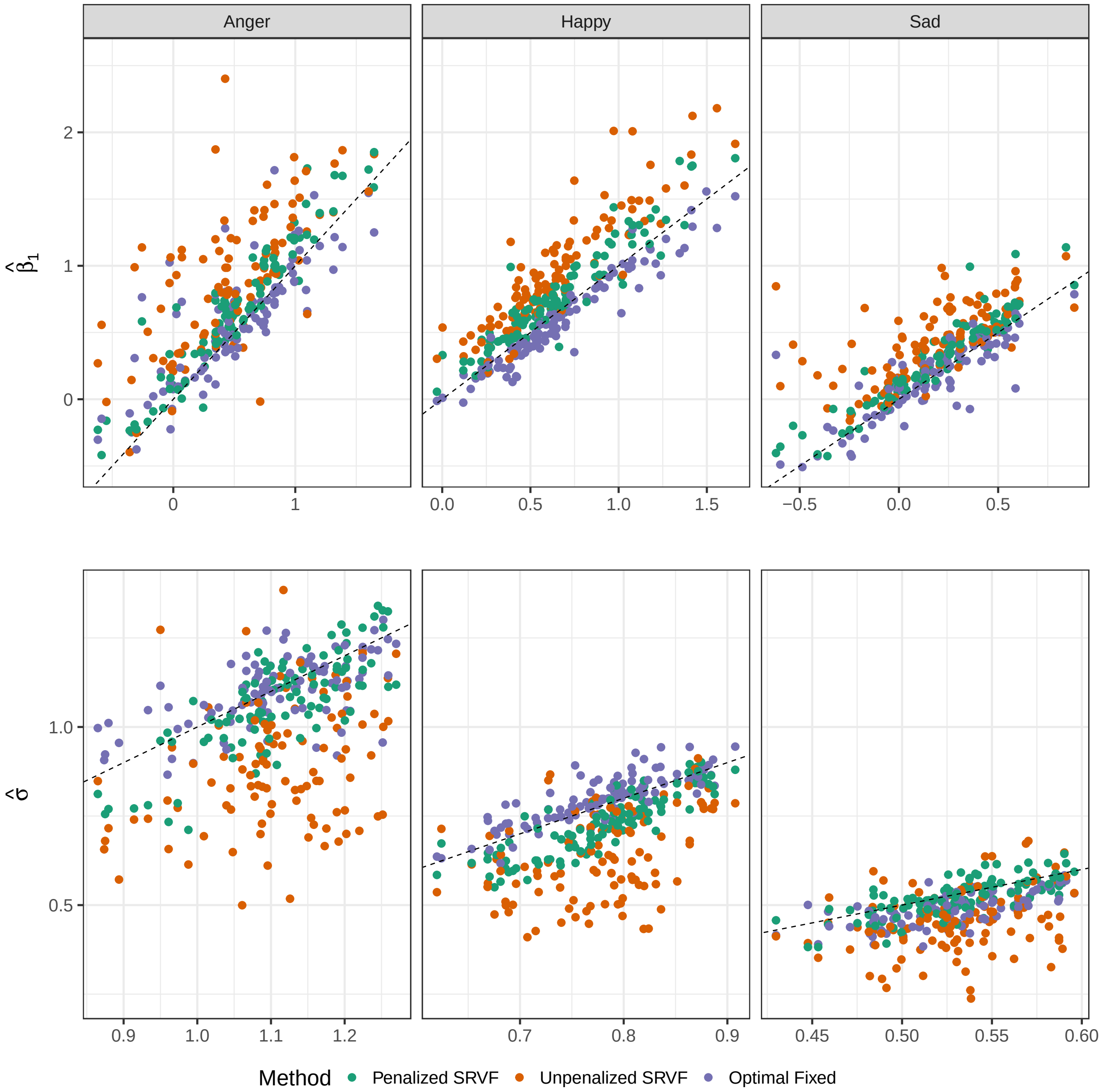

Figure S8: Scatterplots of estimates for the fixed effect discrimination β (top) and random noise standard deviation σ (bottom). In each plot, the horizontal axis represents the estimate when no alignment is conducted, and the vertical axis represents the estimates under unpenalized SRVF (orange) and penalized SRVF with $\nu = 6s$ (green). The dashed line represents the 45 degree line.

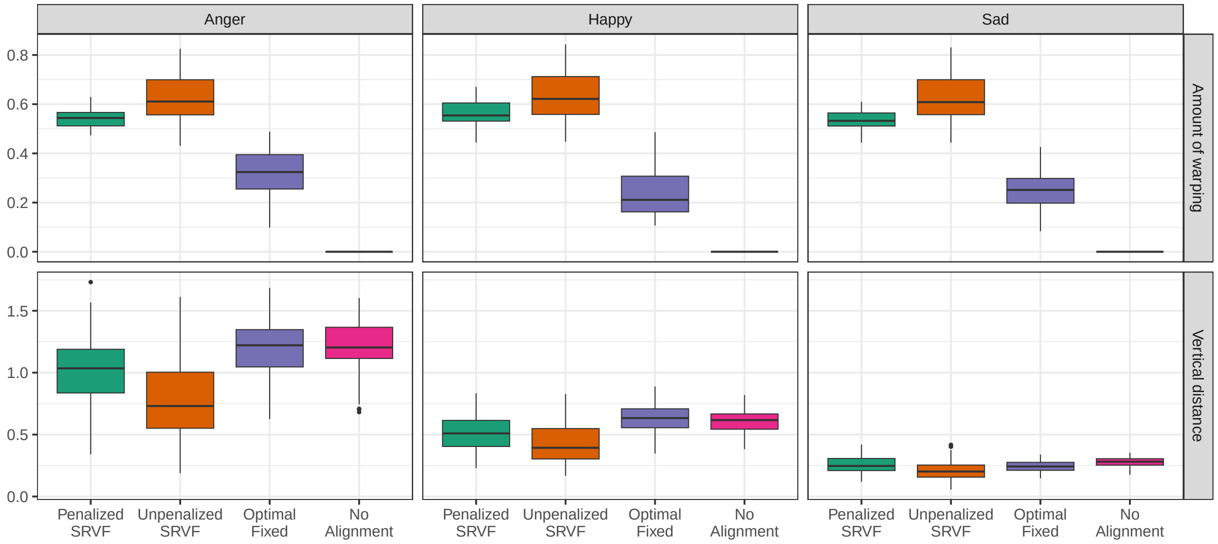

Figure S9: Boxplots of the vertical distance and the average amount of warpings of the estimated models for the three sets of music recordings. The penalized SRVF alignment was conducted using the threshold $\nu = 10s$.

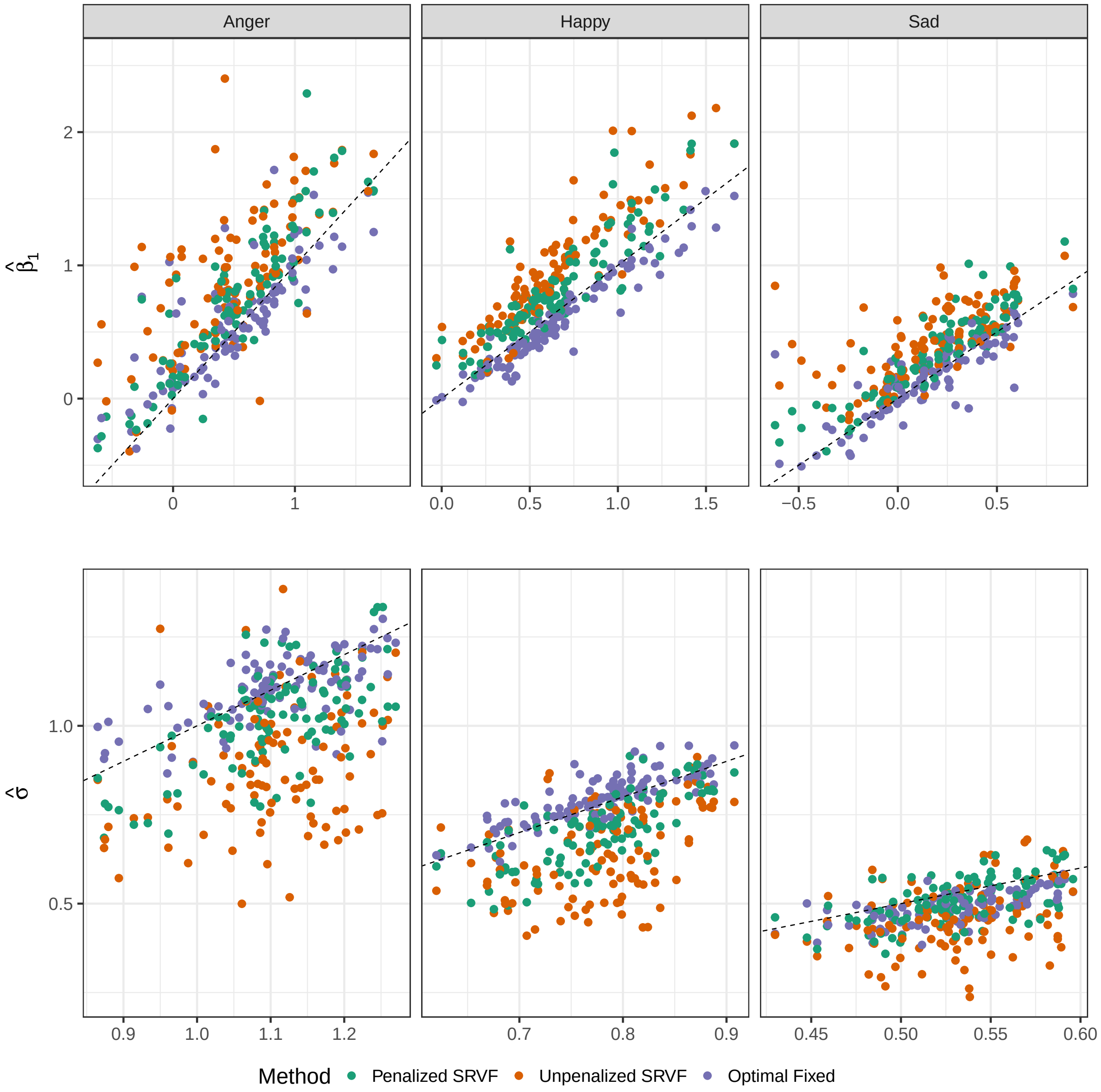

Figure S10: Scatterplots of estimates for the fixed effect discrimination β (top) and random noise standard deviation σ (bottom). In each plot, the horizontal axis represents the estimate when no alignment is conducted, and the vertical axis represents the estimates under unpenalized SRVF (orange) and penalized SRVF with $\nu = 10s$ (green). The dashed line represents the 45 degree line.

Table S5: Mean (standard deviation) of parameter estimates across 113 perceivers in the music empathy study using different alignment methods, including no alignment, unpenalized SRVF, and penalized SRVF with thresholds $\nu \in \{6, 8, 10\}$ seconds.

	Parameter	No alignment	Unpenalized SRVF	Penalized SRVF $\nu = 6$	$\nu = 8$	$\nu = 10$
Anger	Amount of warping	0.00 (0.00)	0.63 (0.09)	0.51 (0.03)	0.53 (0.03)	0.54 (0.04)
	Vertical distance	1.19 (0.24)	0.80 (0.33)	1.10 (0.33)	1.06 (0.32)	1.01 (0.31)
	β_0	0.52 (3.75)	-1.32 (4.02)	-0.26 (4.12)	-0.33 (4.11)	-0.51 (4.08)
	β_1	0.64 (0.81)	1.08 (0.85)	0.83 (0.89)	0.85 (0.89)	0.89 (0.87)
	σ	1.09 (0.12)	0.88 (0.19)	1.04 (0.17)	1.02 (0.17)	1.00 (0.16)
	σ_a	3.58 (3.10)	3.63 (3.06)	3.94 (3.40)	3.92 (3.24)	3.98 (3.32)
	σ_b	0.81 (0.64)	0.84 (0.62)	0.89 (0.69)	0.89 (0.66)	0.91 (0.67)
Happy	Amount of warping	0.00 (0.00)	0.64 (0.10)	0.52 (0.04)	0.55 (0.05)	0.56 (0.05)
	Vertical distance	0.60 (0.10)	0.42 (0.16)	0.53 (0.13)	0.52 (0.14)	0.51 (0.14)
	β_0	2.08 (2.95)	-0.27 (4.28)	1.22 (2.99)	1.03 (2.99)	0.92 (3.07)
	β_1	0.74 (0.53)	1.13 (0.80)	0.87 (0.55)	0.90 (0.55)	0.91 (0.56)
	σ	0.77 (0.07)	0.64 (0.12)	0.72 (0.09)	0.71 (0.10)	0.71 (0.10)
	σ_a	2.16 (1.89)	3.02 (2.87)	2.17 (1.78)	2.26 (1.79)	2.43 (2.00)
	σ_b	0.34 (0.35)	0.50 (0.59)	0.35 (0.33)	0.37 (0.32)	0.39 (0.37)
Sad	Amount of warping	0.00 (0.00)	0.63 (0.09)	0.50 (0.04)	0.52 (0.04)	0.53 (0.04)
	Vertical distance	0.28 (0.04)	0.21 (0.08)	0.27 (0.06)	0.26 (0.07)	0.26 (0.07)
	β_0	3.10 (1.91)	2.13 (3.40)	2.61 (2.00)	2.47 (2.04)	2.35 (2.08)
	β_1	0.21 (0.41)	0.43 (0.69)	0.32 (0.42)	0.36 (0.43)	0.38 (0.44)
	σ	0.53 (0.04)	0.45 (0.09)	0.52 (0.06)	0.51 (0.06)	0.50 (0.07)
	σ_a	2.49 (2.51)	2.85 (5.17)	2.60 (3.01)	2.62 (3.00)	2.70 (3.21)
	σ_b	0.49 (0.50)	0.56 (1.04)	0.51 (0.61)	0.51 (0.61)	0.53 (0.65)
